# Age, Gender, and BMI Modulate the Hepatotoxic Effects of Brominated Flame Retardant Exposure in US Adolescents and Adults: A Comprehensive Analysis of Liver Injury Biomarkers

**DOI:** 10.3390/toxics12070509

**Published:** 2024-07-15

**Authors:** Tingting Li, Wanjing Xu, Yue Zhang, Xueman Ding, Li Liu, Panpan Xu, Hengrui Xing, Yue Ma, Mulatibieke Keerman, Qiang Niu

**Affiliations:** 1 Department of Preventive Medicine, School of Medicine, Shihezi University, Shihezi 832000, China; litingting@stu.shzu.edu.cn (T.L.); 2024009@qzmc.edu.cn (W.X.); 20222014116@stu.shzu.edu.cn (Y.Z.); dingxueman@stu.shzu.edu.cn (X.D.); 20222114168@stu.shzu.edu.cn (L.L.); xupanpan@stu.shzu.edu.cn (P.X.); xinghengrui@stu.shzu.edu.cn (H.X.); ma_yue@stu.shzu.edu.cn (Y.M.); 2 Key Laboratory of Xinjiang Endemic and Ethnic Diseases (Ministry of Education), School of Medicine, Shihezi University, Shihezi 832000, China; 3 NHC Key Laboratory of Prevention and Treatment of Central Asia High Incidence Diseases, First Affiliated Hospital, School of Medicine, Shihezi University, Shihezi 832000, China

**Keywords:** BFRs, liver injury, demographic modifiers, combined exposure, toxicity biomarkers

## Abstract

Brominated flame retardants (BFRs), commonly found in consumer products, have been identified as potential hazards to liver function. While the individual effects of specific BFRs are somewhat understood, there is limited evidence on how mixtures of these chemicals, especially when influenced by demographic factors, interact to affect liver function. This study utilized data from 10,828 participants aged 12 and above from the National Health and Nutrition Examination Survey (2005–2016) to investigate the associations between BFRs (both individually and in combinations) and biomarkers of liver injury. The study focused on how age, gender, and body mass index (BMI) modify modulate these effects. Multivariate linear regression, restricted cubic spline function, weighted quantile sum (WQS) regression, and quantile g-computation (qgcomp) models were used to analyze the linear, non-linear, and joint associations between BFR levels and liver function parameters. We found positive associations between the mixed BFRs index and AST, ALT, GGT, ALP, and TBIL levels and a negative association with ALB levels. PBDE28, PBDE47, and PBB153 consistently contributed to the top weight in both the WQS and qgcomp models. Most critically, the study demonstrated that the relationship between co-exposure to BFRs and liver function parameters was modified by age, gender, and BMI. Therefore, our study highlights the importance of considering demographic diversity in assessing the risk of BFR-induced liver damage and supports the implementation of tailored preventive and intervention strategies.

## 1. Introduction

Brominated flame retardants (BFRs) are commonly used as additives in products such as electronics, furniture, and textiles [1,2] and have become a global concern due to their long-term effects on the environment and health. Although international regulations, particularly the Stockholm Convention, imposed strict restrictions or bans on certain BFRs [3,4], these chemicals still exist in the environment, soil, water, and even in the human body [5,6] due to their extensive use in the past, forming a permanent “chemical footprint”. The accumulation of this persistent organic pollutant poses ongoing challenges to ecosystems and human health, especially for vulnerable populations such as children and pregnant women [7,8,9].

The liver, as the main site of detoxification, metabolism, and nutrient storage [10], has become the focus of environmental health research due to its sensitivity to environmental pollutants and critical functions [11]. Previous studies found an association between BFR exposure and changes in liver function. A cross-sectional study showed that BFR levels in hair or nails were positively correlated with liver parameters such as serum total protein (TP) and serum total bilirubin (TBIL), suggesting that higher BFR concentrations are associated with elevated liver biomarkers (r*_TP_* = 0.267, *p* = 0.033; r*_TBIL_* = 0.325, *p* = 0.047) [12]. Likewise, studies of blood samples showed similar associations between BFR and elevated TBIL (r*_TBIL_* = 0.190, *p* = 0.014) as well as other indicators of liver function [13]. Experimental models demonstrated that prolonged exposure to 2,2′,4,4′-tetrabromodiphenyl ether (PBDE47) at a dosage of 0.2 mg/kg resulted in a reduction in the activity of fatty acid translocase (FAT)/CD36 in liver cells [14]. Exposure to PBDE47 was observed to exacerbate weight gain and liver fat accumulation along with increasing inflammatory responses in male C57BL/6J mice that were consuming a high-fat diet [15].

While previous studies have provided insights into the hepatic effects of individual BFR exposure, real-life scenarios often involve simultaneous exposure to a variety of chemicals that can interact in complex ways [16]. However, these individual studies do not fully capture the intricate nature of human exposure to ‘chemical cocktails’, making it difficult to evaluate the related health risks [17]. Therefore, it is imperative to explore the effects of concurrent exposure to different BFRs on liver health. This necessitates a shift in research methodology, moving from only a narrow focus on single chemicals towards a more holistic approach that considers the synergistic effects of multiple BFRs. Understanding the cumulative impact on liver function is crucial for a comprehensive assessment of the health risks associated with real-world BFR exposure scenarios.

Individual differences, such as age, gender, and body mass index (BMI), impact the in vivo behavior and toxicological effects of chemicals. Age is particularly crucial in liver development and detoxification capacity, making children more susceptible to chemicals due to their less mature hepatic function. Gender differences can lead to diverse endocrine responses, affecting liver metabolism and the elimination of BFRs [18]. Additionally, BMI, which reflects obesity, is closely associated with BFR accumulation in adipose tissue, potentially exacerbating hepatotoxic effects [19]. Although some studies have investigated the liver damage associated with BFRs, there remains a lack of comprehensive research on how gender, age, and BMI influence the relationship between BFR exposure and liver damage, especially in terms of multiple BFR exposures.

This study analyzed data extracted from the National Health and Nutrition Examination Survey (NHANES) between 2005 and 2016, focusing on American adolescents and adults aged 12 years and above. Statistical methods including the weighted linear regression, restricted cubic spline (RCS), weighted quantiles sum regression (WQS), and quantile g-equation (qgcomp) methods were used to explore the connections between individual and combined exposures to BFRs and biomarkers of liver injury (such as alanine aminotransferase (ALT), aspartate transaminase (AST), gamma-glutamyl transferase (GGT), alkaline phosphatase (ALP), albumin (ALB), TBIL, and TP). The study also investigated the influence of gender, age, and BMI on BFR exposure and liver injury through stratified analyses. These efforts provide a more precise foundation for enhancing health risk assessments and targeted interventions.

## 2. Materials and Methods

### 2.1. Participants

Our study employed data obtained from the NHANES, an annually administered, nationwide cross-sectional assessment conducted by the National Center for Health Statistics at the Centers for Disease Control and Prevention (CDC). The NHANES employs a sophisticated, multistage probability design to sample the civilian, non-institutionalized population across the 50 states and DC [20]. Household participation is initiated through email invitations to complete an online questionnaire to assess eligibility. Once deemed eligible, individuals are contacted for a phone interview and subsequently scheduled for a health screening at an NHANES Mobile Health Screening Center [21]. This comprehensive survey thoroughly evaluates the health and nutritional condition of the American population. Adhering strictly to the ethical guidelines stipulated by the National Center for Health Statistics’ (NCHS) Research Ethics Review Board, the study ensured the procurement of informed consent from all participants.

We selected participants aged 12 years and above with complete serum BFR and liver function parameter data from six cycles of the NHANES (2005–2016) [22]. The exclusion criteria were as follows: (1) participants who tested positive for hepatitis B or C (N = 1129); (2) missing serum BFR data or liver parameters (N = 681); (3) missing data for covariates (N = 1118). After applying these criteria, a final sample of 10,828 participants was included in the study. The systematic participant selection method is illustrated in Figure S1.

### 2.2. BFR Measure

Blood samples were collected from participants aged 12 years and older by a phlebotomist at the mobile examination centers (MECs). After collection, the samples were promptly frozen at −20 °C and then transported to the Division of Environmental Health Laboratory Sciences, located within the National Center for Environmental Health at the CDC, for analysis. The NHANES database analyzed eleven polybrominated diphenyl ethers (PBDEs) and PBB153 in serum using automated liquid/liquid extraction and subsequent sample clean-up. The final determination of target analytes was conducted through isotope dilution gas chromatography high-resolution mass spectrometry (GC/IDHRMS) [23]. For BFR levels that fell below the detection limit, the values were expressed by dividing the detection limit by the square root of 2, as per standard methodology [24]. The detection rate was calculated as the number of participants with a detection value above the limit divided by the total population. A list of abbreviations for BFRs can be found in Table S1.

### 2.3. Liver Function Biomarkers

Fasting blood samples were collected from NHANES participants aged 12 years and older at a mobile screening center. The samples were refrigerated and transported to a central laboratory where indicators of liver function tests (LFTs) were measured using the DxC800 Synchron Clinical System (Beckman Coulter, Brea, CA, USA). The severity of liver injury was assessed using serum AST, ALT, GGT, ALP, ALB, TBIL, and TP, which are commonly used to evaluate liver function. The DxC800 system measures serum AST, ALT, GGT, and ALP activity using an enzyme rate method; refrigerated serum ALB using a bichromatic digital endpoint method; serum TBIL using a timed-endpoint Diazo method (Jendrassik-Grof); and serum TP using a timed rate biuret method [25].

### 2.4. Covariates

Covariates were obtained from the NHANES database, including gender, age, race/ethnicity, weight status (BMI), poverty-to-income ratio (PIR), serum creatinine level, smoking status, six-month time period when surveyed, and time of blood draw. Age, gender (male and female), race (Mexican American, other Hispanic, non-Hispanic white, non-Hispanic black, other race, and multiracial), BMI (<25 kg/m^2^ and ≥25 kg/m^2^), PIR (<1 and ≥1), six-month time period when surveyed (November 1 to April 30 and May 1 to October 31), and time of blood draw (morning, afternoon, and evening) were obtained during a household interview which was conducted in-person with an interviewer. Serum cotinine was measured by an isotope-dilution high-performance liquid chromatography/atmospheric pressure chemical ionization tandem mass spectrometric method. Creatinine was measured using the DxC800 Synchron Clinical System.

### 2.5. Statistical Analyses

For descriptive analyses, continuous variables are expressed as the data mean (SD) or median (P_25_, P_75_), calculated using weighted data, and categorical variables are expressed as count (%), with the count calculated using unweighted data and the percentage calculated using weighted data. Spearman correlation analysis was used to assess the correlation between the concentrations of BFRs. As the levels of serum BFRs and liver function indices were skewed, they were transformed by natural logarithm (ln) to improve the normality of the data.

Weighted linear regression and RCS were used to assess the effects of single BFR exposure on indicators of LFTs, and beta coefficients, 95% CI, and *p*-values were calculated for the correlation between each BFR and liver function indicators. In addition, according to the quartiles of single serum BFR concentrations, the study subjects were divided into four groups, and BFRs were included as categorical variables in the regression model with the lowest quartile as the reference in order to analyze the correlation between the different concentration groups and liver function indices and to explore the dose–response relationship (*p* for trend). RCS was used to assess the nonlinear relationship between BFR exposure alone and indicators of LFTs.

WQS and qgcomp models were used to analyze the effect of combined exposures on indicators of LFTs. WQS regression allows estimation of the overall exposure burden values for a population. By using different chemicals as ordinal variables (quartiles), the WQS regression model computes a weighted linear index representing the whole-body burden of a series of chemicals. We used WQS regression models to analyze the effect of combined exposures on indicators of LFTs. It was also possible to analyze the magnitude of the weights that play a role in the combined exposure process of these chemicals. We randomly divided the data into two datasets, with 40% used as the training set and 60% as the validation set, seed = 2016, and the coefficients of the WQS indices were set as positive coefficients to obtain weighted linear indices in the same direction of change of liver function indices and levels of BFRs, as well as the weights for each of the BFRs. The WQS coefficients were then constrained to negative coefficients to determine whether there was a correlation in that direction. Qgcomp modeling, on the other hand, is a method that has recently become widely used in environmental epidemiology for estimating the effects of exposure mixtures [26]. Qgcomp evaluates the combined effect of raising one quartile across all exposures without the assumption of directional homogeneity.

In addition, stratified analyses were performed for age, sex, and BMI. In the weighted linear regression and WQS models, the interaction term was the product of the categorical variable and the level of BFRs.

### 2.6. Sensitivity Analyses

Considering the established impact of alcohol consumption on liver function and the limitations in obtaining comprehensive data on adolescent drinking practices, our study incorporated alcohol consumption as a variable in all regression analyses for participants aged 18 years and above. This inclusion aimed to elucidate the potential modifying effect of alcohol use on the hepatotoxic implications of exposure to BFRs within this population segment. Adjusted confounders included gender, age, race, PIR, drinking status, cotinine levels (as a nicotine metabolite indicating smoking status), the time of blood sample collection, and the six-month period during which the survey was conducted. Such comprehensive adjustments ensure the robustness of the findings, facilitating a more accurate dissection of the complex interplay between BFR exposure, alcohol intake, and liver health in the adult population.

In this study, a significance level of 0.05 is specified, and all analyses were performed in R (version 4.2.1) software using the R packages “ggrcs” (version 0.2.7), “g WQS” (version 3.0.4), and “qgcomp” (version 2.10.1).

## 3. Results

### 3.1. Demographic Characteristics

Table 1 presents the general characteristics of the sex-stratified study population, comprising a total of 10,828 participants, with 5236 males and 5592 females. The average age of the total population was 43.00 years, with males having a mean age of 42.00 years and females, 43.90 years. The detection rates for various BFRs are detailed in Table 2. BFRs with detection rates >90% were considered, including PBDE28, PBDE47, PBDE99, PBDE100, PBDE153, and PBB153. The BFRs exhibited varying degrees of correlation with each other (r*_s_* range: 0.17~0.93), with the strongest correlation observed between PBDE47 and PBDE99 (r*_s_* = 0.93, *p* < 0.01), as depicted in Table S2.

### 3.2. Associations of Individual BFR Exposure with LFTs

**Figure 1:**
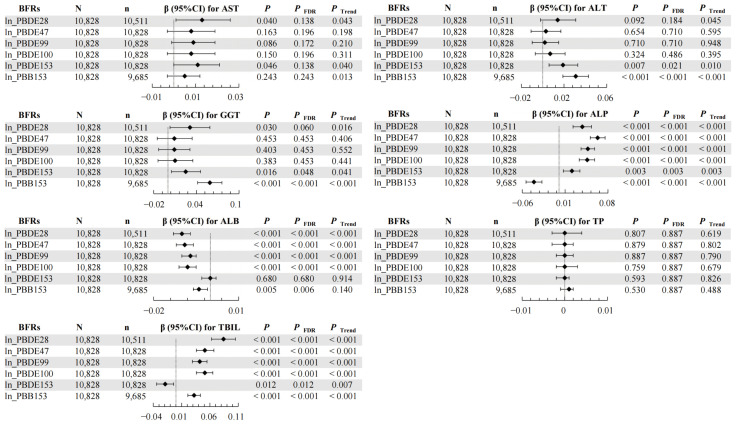
Associations of serum BFRs with LFTs. The model was adjusted by gender (male, female), age (continuous), race (Mexican American, Other Hispanic, Non-Hispanic White, Non-Hispanic Black, Other Race—including multi-racial), BMI (<25 kg/m^2^ and ≥25 kg/m^2^), PIR (<1 and ≥1), creatinine (continuous), cotinine (continuous), time of blood draw (morning, afternoon, evening), and six-month time period when surveyed (November 1 through April 30, May 1 through October 31). “N” means total number. “n” means the number of BFR-positive subjects. “◆” means β value and line segment means 95%CI. All liver function indicator variables were ln-transformed before analysis.

The impact of each BFR on LFTs is illustrated in Figure 1. After adjusting for potential confounding variables, positive correlations were observed between PBDE153 (β = 0.019; 95% CI: 0.006, 0.033) and PBB153 (β = 0.031; 95% CI: 0.019, 0.043) and ALT levels. Similarly, PBDE153 (β = 0.025; 95% CI: 0.005, 0.046) and PBB153 (β = 0.059; 95% CI: 0.042, 0.076) exhibited positive correlations with GGT. PBDE28 (β = 0.038; 95% CI: 0.023, 0.053), PBDE47 (β = 0.063; 95% CI: 0.051, 0.075), PBDE99 (β = 0.047; 95% CI: 0.036, 0.058), PBDE100 (β = 0.046; 95% CI: 0.033, 0.058), and PBDE153 (β = 0.021; 95% CI: 0.007, 0.034) were positively associated with ALP, while PBB153 (β = −0.041; 95% CI: −0.054, −0.028) showed a negative correlation. PBDE28, PBDE47, PBDE99, PBDE100, and PBB153 demonstrated positive correlations with TBIL, whereas PBDE153 displayed a negative correlation (β = −0.019; 95% CI: −0.034, −0.004). All correlations followed a dose–response pattern (*p* for trend < 0.05) (Tables S3–S9).

### 3.3. Associations of Combined BFRs Exposure with LFTs

The study examined the combined impact of BFR mixtures on liver injury using the WQS and qgcomp models. In the WQS model, combined exposure to BFRs was found to have a positive association with AST (β = 0.010; 95% CI: 0.001, 0.019), ALT (β = 0.050; 95% CI: 0.035, 0.066), GGT (β = 0.074; 95% CI: 0.054, 0.093), ALP (β = 0.038; 95% CI: 0.029, 0.047), and TBIL (β = 0.044; 95% CI: 0.033, 0.055) and a negative association with ALB (β = −0.007; 95% CI: −0.009, −0.005) (Table 3). The BFR weights in the WQS model, illustrated in Figures S9 and S10, highlighted PBDE28, PBDE47, and PBB153 as the most influential compounds.

Similarly, in the qgcomp model, co-exposure to BFRs showed a positive correlation with ALT (β = 0.036; 95% CI: 0.019, 0.053), GGT (β = 0.075; 95% CI: 0.050, 0.100), and TBIL (β = 0.035; 95% CI: 0.019, 0.051) and a negative correlation with ALB (β = −0.006; 95% CI: −0.009, −0.003) (Table 2). The BFR weights in the qgcomp model, shown in Figure S11, also identified PBDE28, PBDE47, and PBB153 as the main contributors, aligning with the findings from the WQS model.

### 3.4. Modification Effects of Age, Gender, and BMI on the Association between BFRs and LFTs

**Figure 2:**
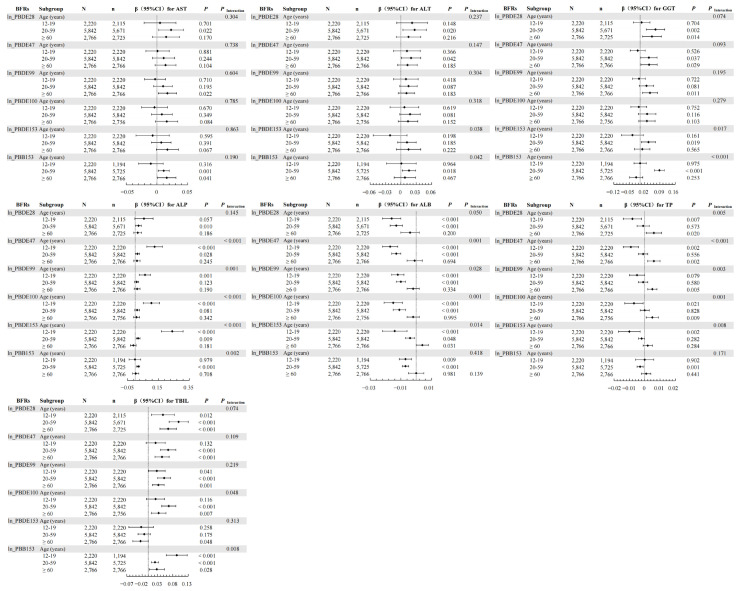
Associations of serum BFRs with LFTs stratified by age. The model was adjusted by gender (male, female), race (Mexican American, Other Hispanic, Non-Hispanic White, Non-Hispanic Black, Other Race—including multi-racial), BMI (<25 kg/m^2^ and ≥25 kg/m^2^), PIR (<1 and ≥1), creatinine (continuous), cotinine (continuous), time of blood draw (morning, afternoon, evening), and six-month time period when surveyed (November 1 through April 30, May 1 through October 31). “N” means total number. “n” means the number of BFR-positive subjects. “◆” means β value and line segment means 95%CI.

**Figure 3:**
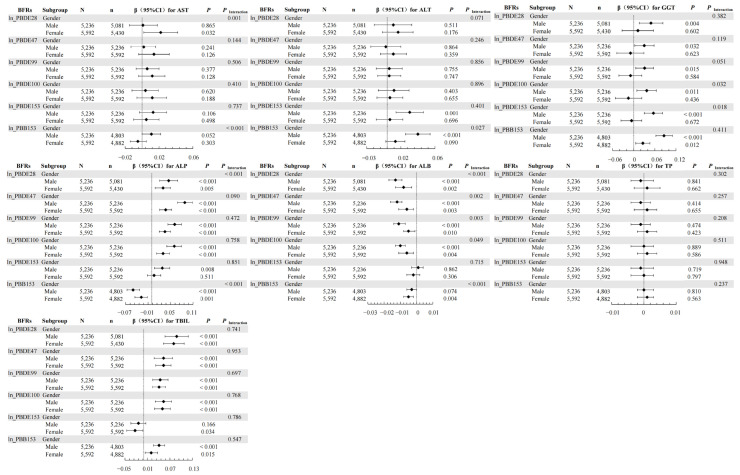
Associations of serum BFRs with LFTs stratified by gender. The model was adjusted by age (continuous), race (Mexican American, Other Hispanic, Non-Hispanic White, Non-Hispanic Black, Other Race—including multi-racial), BMI (<25 kg/m^2^ and ≥25 kg/m^2^), PIR (<1 and ≥1), creatinine (continuous), cotinine (continuous), time of blood draw (morning, afternoon, evening), and six-month time period when surveyed (November 1 through April 30, May 1 through October 31). “N” means total number. “n” means the number of BFR-positive subjects. “◆” means β value and line segment means 95%CI.

**Figure 4:**
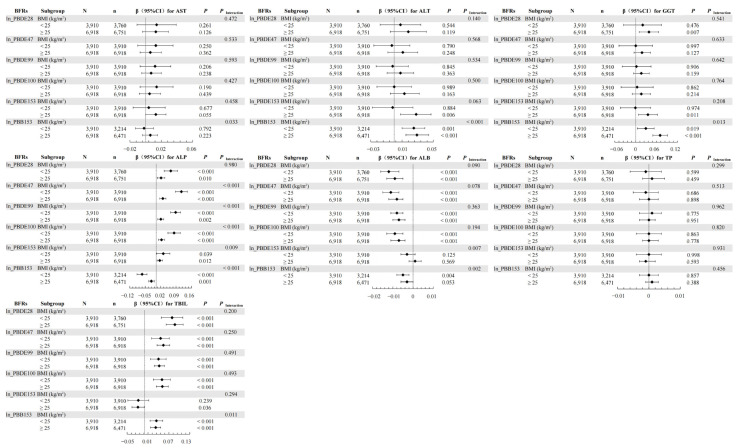
Associations of serum BFRs with LFTs stratified by BMI. The model was adjusted by gender (male, female), age (continuous), race (Mexican American, Other Hispanic, Non-Hispanic White, Non-Hispanic Black, Other Race—including multi-racial), PIR (<1 and ≥1), creatinine (continuous), cotinine (continuous), time of blood draw (morning, afternoon, evening), and six-month time period when surveyed (November 1 through April 30, May 1 through October 31). “N” means total number. “n” means the number of BFR-positive subjects. “◆” represents β value and line segment means 95%CI.

In the weighted linear regression, interactions were observed between BFRs and age groups for ALT, GGT, ALP, ALB, and TBIL (Figure 2); between BFRs and gender for AST, ALT, GGT, ALP, and ALB (Figure 3); and between BFRs and BMI for AST, ALT, ALP, ALB, and TBIL (Figure 4).

The WQS model showed that combined exposure interacted with age subgroups for ALP and ALB (Table 4) and with gender subgroups for AST, ALT, ALP, and ALB (Table 5). Notably, co-exposure did not correlate with TP in the overall population but had a negative correlation with TP in males and no correlation in females, indicating a gender-specific interaction. Additionally, there was an interaction between combined exposure and BMI subgroups for ALT, GGT, ALP, and TBIL (Table 6).

In the qgcomp model, age-stratified analyses revealed correlations mainly in individuals aged 20–59 years (Table S10). Gender-stratified analyses showed that co-exposure was positively associated with AST, ALT, GGT, and TBIL and negatively associated with ALB in males, while in females, co-exposure was positively correlated with TBIL and negatively correlated with ALB (Table S11). Furthermore, BMI-stratified analyses indicated that co-exposure was positively associated with ALT, GGT, and TBIL and negatively associated with ALP and ALB in individuals with BMI < 25 kg/m^2^, while it was positively associated with AST, ALT, GGT, and TBIL and negatively associated with ALB in those with BMI ≥ 25 kg/m^2^ (Table S12).

### 3.5. Sensitivity Analyses

Incorporating the variable of alcohol consumption into the regression models assessing the relationships with liver function biomarkers, such as AST, ALT, GGT, ALP, TP, and TBIL, did not significantly change the statistical significance of the observed correlations, except for ALP. Notably, the addition of alcohol intake resulted in a statistically significant change in the association with ALP (refer to Tables S13 and S14).

## 4. Discussion

The study found relationships between individual and combined exposures to brominated flame retardants (BFRs) and liver function tests (LFTs), which were influenced by age, gender, and BMI. Specifically, PBDE28, PBDE47, and PBB153 were identified as important components in combined exposures, showing positive correlations with elevated levels of various liver function parameters and a negative correlation with ALB. Regardless of the analytical method used (weighted linear regression, RCS, WQS, or qgcomp model), exposure to BFRs was associated with increased levels of ALT, GGT, and TBIL, along with decreased ALB concentrations. These effects differed based on gender, BMI category, and age, underscoring the importance of considering these factors when assessing health risks associated with BFRs.

Enzymes such as AST and ALT are typically contained within liver cells, but in cases of hepatocellular damage or necrosis, they leak into the bloodstream, serving as clinical markers for liver dysfunction [8,27,28]. Our study revealed a substantial positive correlation between combined exposure to BFRs and elevated AST and ALT levels. Supporting evidence from research on endocrine-disrupting compounds, which outlined the delineation of liver injury risk at AST and ALT values surpassing the 90th percentile for the population examined, showed that polybrominated diphenyl ethers (PBDEs) have been associated with an increased odds ratio of 1.57 (CI = 1.34, 1.84) for hepatic injury occurrence [29], which is consistent with our findings.

ALP levels notably escalate in cases of biliary obstruction [30] and serve as an indicator of hepatic dysfunction [31]. Moreover, GGT, found in liver tissue, helps differentiate elevated ALP levels of liver origin from those derived from bones, confirming liver-related issues and aiding in the detection of cholestatic liver diseases. Our study also found a positive correlation between exposure to BFRs and increased ALP and GGT levels. Additionally, a rodent study revealed that BFR exposure during prenatal and early postnatal stages led to an increase in liver mass among progeny, and notably, male offspring exhibited a significant surge in ALP levels [32].

TP levels reflect the liver’s capacity to synthesize and store proteins, with ALB being a protein uniquely produced by the liver and essential for various physiological functions. Due to ALB’s exclusive origin in the liver, its concentration is commonly used to evaluate liver synthetic efficiency. Our study identified a negative correlation between simultaneous exposure to BFRs and ALB levels. In a study conducted by Van den Steen and colleagues [33], female European starlings exposed to PBDEs displayed a reduction in ALB concentrations after short exposure durations (as shown by LSD test with *p* = 0.07). Importantly, this decline in ALB was not accompanied by changes in TP levels, suggesting that short-term PBDE exposure may specifically result in decreased ALB concentrations.

Bilirubin is transported to the liver in an insoluble state initially, where it is converted into a soluble form for elimination. Elevated TBIL levels indicate disrupted hepatic metabolic processes [27]. In the present study, a positive correlation was observed between TBIL levels and exposure to BFRs. Echoing these findings, a cross-sectional study in China reported a significant positive relationship between nail concentrations of PBDE209 and TBIL (r*_TP_* = 0.267, *p* = 0.033; r*_TBIL_* = 0.325, *p* = 0.047) [12]. Moreover, another cross-sectional study in the main BFR-polluted region of Shandong, China, found a positive correlation between serum BFR concentrations and hepatic function markers, including TBIL (r*_TBIL_* = 0.190, *p* = 0.014) [13].

Investigating the relationship between BFRs and hepatic function has revealed age-related disparities, gender-specific effects, and BMI-dependent responses. In age-stratified analyses, the application of weighted linear regression, the WQS model, and the qgcomp method illuminated that BFRs exert a spectrum of age-specific influences on hepatic function. For individuals aged 12–19, no significant correlation was identified between individual or combined BFR exposure and levels of AST, ALT, and GGT, a finding potentially attributed to the restricted sample size within this age category. Moreover, the primary route of BFR biotransformation in the liver occurs through the CYP450 oxidative system [34]. Hepatic drug metabolism capabilities evolve with age, as evidenced by marked changes in CYP gene expression profiles in the liver [35]. The gender-segregated studies showed that BFR exposure correlated with AST modifications specifically in females, whereas in males, similar exposure resulted in alterations to ALT, pointing to a gender-specific interaction. These outcomes mirror documented gender differences in responses to hepatotoxic chemicals [36]. Given that BFR-induced liver toxicity potentially arises from CYP450 upregulation, it is important to note that CYP450 isoforms display sexual dimorphism in their expression and activity—men characteristically exhibit higher activity of forms such as CYP1A, while women demonstrate enhanced activity of variants akin to CYP3A [37,38]. This divergent expression might explain the observed variations in BFRs’ impact on the liver according to sex. In our BMI-stratified examination of both singular and combined effects of BFR exposure, the correlation between BFRs and liver function parameters was found to vary, underscoring a BMI-linked interaction. Given BFRs’ lipophilic nature, they are prone to accumulate in adipose tissue, as confirmed by the inverse relationship between serum BFR concentrations and rising BMI [39,40]. This propensity for fat storage could potentially diminish the susceptibility to BFR effects in individuals with higher BMIs. Consequently, a comprehensive understanding of the relationship between gender, BMI, age, and hepatic implications of BFR exposure is crucial.

Previous research has mainly concentrated on the liver effects of individual BFR compounds [12,14], with limited research on the combined impact on liver function [29]. In contrast, our study examined both individual and combined exposures to various BFRs using a large participant group and multiple validation methods to support our findings. Despite the above advantages, our research is not without its constraints. Given alcohol’s known impact on liver function and the challenge of acquiring complete adolescent drinking habit records, we executed a sensitivity analysis that echoed the outcomes of our primary examination. Our cross-sectional approach, assessing serum BFR and liver function markers simultaneously, prevented the establishment of causal relationships. Moreover, a single measurement of serum BFRs may not accurately reflect historic exposure levels. While we adjusted for various confounding factors, there may be unaccounted variables influencing results due to missing data. Further research is necessary to gain a more comprehensive understanding of these associations.

## 5. Conclusions

Utilizing data from the NHANES, our study elucidated important associations between serum BFR exposures, either alone or in combination, and key liver function parameters, while highlighting the key moderating roles of age, sex, and BMI in this intricate relationship. Specifically, our findings revealed that combined exposure to BFRs, with PBDE28, PBDE47, and PBB153 being key contributors, correlates positively with elevated ALT, AST, GGT, ALP, and TBIL, while inversely correlating with ALB. Most importantly, our investigation highlighted the impact of demographic factors on BFR-induced liver effects. Age-related differences in susceptibility patterns; distinct sex-specific responses reflecting variations in the handling of liver function parameters; and the complex interplay with BMI, which implicates adipose tissue accumulation and altered toxicity profiles, collectively paint a nuanced picture of BFRs’ hepatotoxic potential. In summary, our research not only strengthens the evidence for BFRs’ detrimental effects on liver function through both singular and mixed exposures but also accentuates the necessity to consider individual characteristics—age, gender, and BMI—as crucial modifiers in understanding and mitigating BFR-related liver damage. These findings set the stage for more targeted interventions and future studies aimed at deciphering the personalized health risks associated with BFR exposure.

## Figures and Tables

**Table 1:** Characteristics of included participants from the NHANES, 2005–2016 (N = 10,828).

Characteristic	All	Male	Female
N	10,828	5236	5592
Age (years)	43.00 (18.90)	42.00 (18.60)	43.90 (19.10)
Race			
Mexican American	1959 (8.96)	959 (9.98)	1000 (8.02)
Other Hispanic	1005 (5.31)	456 (5.31)	549 (5.32)
Non-Hispanic White	4469 (67.79)	2204 (67.96)	2265 (67.64)
Non-Hispanic Black	2356 (11.02)	1133 (10.08)	1223 (11.87)
Other Race—including multi-racial	1039 (6.93)	484 (6.68)	555 (7.15)
BMI (kg/m^2^)			
<25	3910 (35.16)	1816 (30.63)	2094 (39.31)
≥25	6918 (64.84)	3420 (69.37)	3498 (60.69)
PIR			
<1	2515 (14.84)	1140 (13.77)	1375 (15.82)
≥1	8313(85.16)	4096 (86.23)	4217 (84.18)
Cotinine, serum (ng/mL)	0.04 (0.01, 2.62)	0.06 (0.02, 26.08)	0.03 (0.01, 0.47)
Creatinine, serum (μmol/L)	73.37 (61.88, 87.52)	83.98 (74.26, 93.70)	65.42 (57.46, 74.26)
Six-month time period			
November 1 through April 30	5208 (42.85)	2486 (42.82)	2722 (42.87)
May 1 through October 31	5620 (57.15)	2750 (57.18)	2870 (57.13)
Time of blood draw			
Morning	5272 (48.47)	2564 (48.99)	2708 (47.99)
Afternoon	3838 (34.38)	1865 (34.07)	1973 (34.67)
Evening	1718 (17.15)	807 (16.95)	911 (17.34)
AST (U/L)	23.00 (20.00, 27.00)	25.00 (21.00, 29.00)	21.00 (18.00, 25.00)
ALT (U/L)	20.00 (16.00, 28.00)	24.00 (19.00, 33.00)	18.00(14.00, 22.00)
GGT (U/L)	18.00 (13.00, 27.00)	22.00 (15.00, 32.00)	15.00 (11.00, 22.00)
ALP (IU/L)	65.00 (53.00, 81.00)	67.00 (55.00, 83.00)	64.00 (52.00, 79.00)
ALB (g/L)	43.00 (41.00, 45.00)	44.00 (42.00, 46.00)	42.00 (40.00, 44.00)
TP (g/dL)	7.10 (6.80, 7.40)	7.10 (6.90, 7.40)	7.00 (6.80, 7.30)
TBIL (mg/dL)	0.70 (0.50, 0.80)	0.70 (0.60, 0.90)	0.60 (0.50, 0.70)

Continuous variables are expressed as mean (SD) or median (P_25_, P_75_), calculated using weighted data; categorical variables are expressed as count (%), with count calculated using unweighted data and the percentage calculated using weighted data.

**Table 2:** The detection rates and distributions of all BFRs.

BFRs (pg/g)	Detection Rates (%)	Positive Subjects (n)	Percentiles		
			P_25_	P_50_	P_75_
PBDE17	6.4	712	0.92	1.27	1.80
PBDE28	97.0	10511	4.50	6.66	10.20
PBDE47	100	10828	79.89	118.30	194.00
PBDE66	12.5	1510	1.20	1.80	1.80
PBDE85	73.4	8479	1.47	2.32	4.10
PBDE99	100	10828	14.62	22.32	38.70
PBDE100	100	10828	15.75	24.36	39.47
PBDE153	100	10828	35.78	55.10	87.78
PBDE154	72.3	8463	1.47	2.09	3.78
PBDE183	39.2	4602	1.13	1.55	1.80
PBDE209	69.3	7400	11.00	17.60	17.70
PBB153	94.2	9685	5.24	13.61	27.13

Positive subjects were participants with detection values above the detection limit. Detection rates (%) were calculated using weighted data, and positive subjects (n) were calculated using unweighted data.

**Table 3:** WQS and qgcomp models’ assessments of the association between combined exposure to serum BFRs and liver function indicators.

	WQS				Qgcomp	
	Positive		Negative			
	β (95%CI)	*p*	β (95%CI)	*p*	β (95%CI)	*p*
AST	0.010 (0.001, 0.019)	0.028	0.009 (−0.001, 0.019)	0.073	0.012 (−0.001, 0.025)	0.069
ALT	0.050 (0.035, 0.066)	<0.001	0.011 (0.001, 0.021)	0.034	0.036 (0.019, 0.053)	<0.001
GGT	0.074 (0.054, 0.093)	<0.001	0.023 (0.008, 0.037)	0.002	0.075 (0.050, 0.100)	<0.001
ALP	0.038 (0.029, 0.047)	<0.001	--		−0.013 (−0.026, 0.001)	0.066
ALB	--		−0.007 (−0.009, −0.005)	<0.001	−0.006 (−0.009, −0.003)	<0.001
TP	0.000 (−0.002, 0.001)	0.635	0.000 (−0.002, 0.003)	0.679	0.001 (−0.002, 0.003)	0.542
TBIL	0.044 (0.033, 0.055)	<0.001	--		0.035 (0.019, 0.051)	<0.001

The model was adjusted by gender (male, female), age (continuous), race (Mexican American, Other Hispanic, Non-Hispanic White, Non-Hispanic Black, Other Race—including multi-racial), BMI (<25 kg/m^2^ and ≥25 kg/m^2^), PIR (<1 and ≥1), creatinine (continuous), cotinine (continuous), time of blood draw (morning, afternoon, evening), and six-month time period when surveyed (November 1 through April 30, May 1 through October 31). “--” means the associations between BFRs and the liver function index were not available in that direction.

**Table 4:** WQS modeling to assess the associations between combined exposure to serum BFRs and indicators of liver function stratified by age.

Indicator	Subgroup	Positive			Negative		
		β (95%CI)	*p*	*p* _Interaction_	β (95%CI)	*p*	*p* _Interaction_
AST	Age (years)			0.282			0.894
	12–19	−0.011 (−0.027, 0.006)	0.210		0.001 (−0.014, 0.016)	0.863	
	20–59	0.026 (0.013, 0.039)	<0.001		0.003 (−0.009, 0.015)	0.583	
	≥60	0.003 (0.016, 0.048)	<0.001		0.018 (0.006, 0.030)	0.003	
ALT	Age (years)			0.020			0.069
	12–19	−0.008 (−0.028, 0.012)	0.444		0.001 (−0.017, 0.019)	0.901	
	20–59	0.021 (0.004, 0.038)	0.016		0.006 (−0.008, 0.021)	0.381	
	≥60	0.012 (−0.006, 0.030)	0.186		0.021 (0.002, 0.041)	0.027	
GGT	Age (years)			<0.001			0.009
	12–19	0.007 (−0.015, 0.030)	0.519		0.001 (−0.026, 0.028)	0.937	
	20–59	0.098 (0.078, 0.119)	<0.001		--		
	≥60	0.030 (0.001, 0.060)	0.044		0.023 (−0.009, 0.055)	0.154	
ALP	Age (years)			<0.001			--
	12–19	0.111 (0.080, 0.141)	<0.001		--		
	20–59	0.022 (0.010, 0.034)	<0.001		0.007 (−0.002, 0.016)	0.129	
	≥60	0.013 (−0.003, 0.030)	0.114		−0.001 (−0.019, 0.016)	0.873	
ALB	Age (years)			--			0.009
	12–19	--			−0.009 (−0.013, −0.005)	<0.001	
	20–59	--			−0.012 (−0.015, −0.009)	<0.001	
	≥60	0.002 (−0.001, 0.006)	0.201		−0.002 (−0.006, 0.002)	0.250	
TP	Age (years)			0.002			0.269
	12–19	−0.002 (−0.006, 0.001)	0.129		−0.005 (−0.008, −0.001)	0.013	
	20–59	−0.001 (−0.003, 0.001)	0.332		−0.004 (−0.006, −0.002)	<0.001	
	≥60	0.004 (0.001, 0.007)	0.007		0.005 (0.002, 0.009)	0.005	
TBIL	Age (years)			0.091			--
	12–19	0.092 (0.071, 0.114)	<0.001		0.010 (−0.016, 0.036)	0.450	
	20–59	0.048 (0.035, 0.060)	<0.001		--		
	≥60	0.044 (0.023, 0.066)	<0.001		−0.027 (−0.044, −0.010)	0.002	

The model was adjusted by gender (male, female), race (Mexican American, Other Hispanic, Non-Hispanic White, Non-Hispanic Black, Other Race—including multi-racial), BMI (<25 kg/m^2^ and ≥25 kg/m^2^), PIR (<1 and ≥ 1), creatinine (continuous), cotinine (continuous), time of blood draw (morning, afternoon, evening), and six-month time period when surveyed (November 1 through April 30, May 1 through October 31). “--” means the associations between BFRs and the liver function index were not available in that direction.

**Table 5:** WQS modeling to assess the associations between combined exposure to serum BFRs and indicators of liver function stratified by gender.

Indicator	Subgroup	Positive			Negative		
		β (95%CI)	*p*	*p* _Interaction_	β (95%CI)	*p*	*p* _Interaction_
AST	Gender			0.005			0.046
	Male	0.006 (−0.010, 0.022)	0.503		−0.003 (−0.014, 0.007)	0.531	
	Female	0.011 (0.001, 0.020)	0.030		0.004 (−0.010, 0.018)	0.536	
ALT	Gender			0.049			0.519
	Male	0.039 (0.017, 0.061)	<0.001		−0.004 (−0.018, 0.010)	0.553	
	Female	0.011 (−0.003, 0.025)	0.117		0.016 (0.000, 0.032)	0.046	
GGT	Gender			0.746			0.002
	Male	0.090 (0.059, 0.120)	<0.001		--		
	Female	0.037 (0.009, 0.065)	0.009		−0.006 (−0.028, 0.016)	0.593	
ALP	Gender			0.035			--
	Male	0.060 (0.045, 0.075)	<0.001		--		
	Female	0.028 (0.017, 0.039)	<0.001		−0.010 (−0.030, 0.009)	0.309	
ALB	Gender			--			< 0.001
	Male	--			−0.011 (−0.013, −0.008)	<0.001	
	Female	−0.003 (−0.006, −0.001)	0.008		−0.006 (−0.010, −0.003)	<0.001	
TP	Gender			0.142			0.041
	Male	−0.003 (−0.006, 0.000)	0.045		−0.003 (−0.005, 0.000)	0.034	
	Female	0.000 (−0.003, 0.003)	0.891		0.002 (0.000, 0.004)	0.077	
TBIL	Gender			0.935			--
	Male	0.050 (0.031, 0.070)	<0.001		--		
	Female	0.044 (0.028, 0.060)	<0.001		--		

The model was adjusted by age (continuous), race (Mexican American, Other Hispanic, Non-Hispanic White, Non-Hispanic Black, Other Race—including multi-racial), BMI (<25 kg/m^2^ and ≥25 kg/m^2^), PIR (<1 and ≥ 1), creatinine (continuous), cotinine (continuous), time of blood draw (morning, afternoon, evening), and six-month time period when surveyed (November 1 through April 30, May 1 through October 31). “--” means the associations between BFRs and the liver function index were not available in that direction.

**Table 6:** WQS modeling to assess the associations between combined exposure to serum BFRs and indicators of liver function stratified by BMI.

Indicator	Subgroup	Positive			Negative		
		β (95%CI)	*p*	*p* _Interaction_	β (95%CI)	*p*	*p* _Interaction_
AST	BMI (kg/m^2^)			0.490			0.354
	<25	0.006 (−0.006, 0.018)	0.305		0.007 (−0.009, 0.023)	0.390	
	≥25	0.015 (0.001, 0.029)	0.039		0.005 (−0.004, 0.014)	0.297	
ALT	BMI (kg/m^2^)			0.002			0.830
	<25	0.029 (0.005, 0.053)	0.017		−0.005 (−0.020, 0.009)	0.479	
	≥25	0.033 (0.014, 0.052)	<0.001		0.002 (−0.010, 0.013)	0.796	
GGT	BMI (kg/m^2^)			0.017			0.383
	<25	0.040 (0.005, 0.074)	0.023		−0.004 (−0.024, 0.016)	0.707	
	≥25	0.071 (0.046, 0.096)	<0.001		0.019 (0.000, 0.038)	0.048	
ALP	BMI (kg/m^2^)			<0.001			--
	<25	0.083 (0.066, 0.100)	<0.001		−0.103 (−0.129, −0.077)	<0.001	
	≥25	0.017 (0.004, 0.030)	0.010		0.010 (−0.004, 0.024)	0.164	
ALB	BMI (kg/m^2^)			--			0.623
	<25	−0.002 (−0.005, 0.001)	0.129		−0.010 (−0.014, −0.006)	<0.001	
	≥25	−0.003 (−0.006, 0.001)	0.097		−0.006 (−0.008, −0.004)	<0.001	
TP	BMI (kg/m^2^)			0.705			0.597
	<25	−0.001 (−0.004, 0002)	0.628		0.000 (−0.004, 0.003)	0.921	
	≥25	0.000 (−0.002, 0.002)	0.990		0.000 (−0.003, 0.002)	0.785	
TBIL	BMI (kg/m^2^)			<0.001			--
	<25	0.053 (0.029, 0.077)	<0.001		--		
	≥25	0.064 (0.049, 0.079)	<0.001		--		

The model was adjusted by gender (male, female), age (continuous), race (Mexican American, Other Hispanic, Non-Hispanic White, Non-Hispanic Black, Other Race—including multi-racial), PIR (<1 and ≥1), creatinine (continuous), cotinine (continuous), time of blood draw (morning, afternoon, evening), and six-month time period when surveyed (November 1 through April 30, May 1 through October 31). “--” means the associations between BFRs and the liver function index were not available in that direction.

## Data Availability

The data were retrieved from publicly available resources and can be accessed from the National Center for Health Statistics of the Center for Disease Control and Prevention through https://www.cdc.gov/nchs/nhanes/index.htm (accessed on 1 March 2023).

## Supplementary Materials

The following supporting information can be downloaded at: https://www.mdpi.com/article/10.3390/toxics12070509/s1, Table S1: The detection rates of all BFRs; Table S2: The Spearman rank correlation coefficients for the concentrations of the BFRs; Table S3: Associations between single BFRs and AST levels based on survey-weighted regression; Table S4: Associations between single BFRs and ALT levels based on survey-weighted regression; Table S5: Associations between single BFRs and GGT levels based on survey-weighted regression; Table S6: Associations between single BFRs and ALP levels based on survey-weighted regression; Table S7: Associations between single BFRs and ALB levels based on survey-weighted regression; Table S8: Associations between single BFRs and TP levels based on survey-weighted regression; Table S9: Associations between single BFRs and TBIL levels based on survey-weighted regression; Table S10: Qgcomp modeling to assess the associations between combined exposure to serum BFRs and indicators of liver function stratified by age; Table S11: Qgcomp modeling to assess the associations between combined exposure to serum BFRs and indicators of liver function stratified by gender; Table S12: Qgcomp modeling to assess the associations between combined exposure to serum BFRs and indicators of liver function stratified by BMI; Table S13: Associations between single BFRs with LFTs based on survey-weighted regression; Table S14: WQS and qgcomp models to assess the association between combined exposure to serum BFRs and LFTs; Figure S1: Flow chart of study population exclusion criteria (N = 10828), NHANES 2005–2016; Figure S2: Nonlinear correlation between serum BFR concentration and AST levels; Figure S3: Nonlinear correlation between serum BFR concentration and ALT levels; Figure S4: Nonlinear correlation between serum BFR concentration and GGT levels; Figure S5: Nonlinear correlation between serum BFR concentration and ALP levels; Figure S6: Nonlinear correlation between serum BFR concentration and ALB levels; Figure S7: Nonlinear correlation between serum BFR concentration and TP levels; Figure S8: Nonlinear correlation between serum BFR concentration and TBIL levels; Figure S9: Weight of each BFR in the positive WQS model (Positive). Figure S10: Weight of each BFR in the negative WQS model (Negative); Figure S11: Weight of each BFR metabolite in the qgcomp model.
