# Supplementary material for: Age, Gender, and BMI Modulate the Hepatotoxic Effects of Brominated Flame Retardant Exposure in US Adolescents and Adults: A Comprehensive Analysis of Liver Injury Biomarkers
Source: Toxics. 2024 Jul 15;12(7):509. doi: 10.3390/toxics12070509 (PMC11280492; doi:10.3390/toxics12070509)
Supplement: Supplementary file 1 [file toxics-12-00509-s001.zip › Table S1.pdf]

Table S1 List of abbreviations for BFRs.

Abbreviations	Applied Chemistry name
PBDE17	2,2´,4-tribromodiphenyl ether
PBDE28	2,4,4´-tribromodiphenyl ether
PBDE47	2,2´,4,4´-tetrabromodiphenyl ether
PBDE66	2,3´,4´,4-tetrabromodiphenyl ether
PBDE85	2,2´,3,4,4´-tentabromodiphenyl ether
PBDE99	2,2´,4,4´,5-pentabromodiphenyl ether
PBDE100	2,2´,4,4´,6-pentabromodiphenyl ether
PBDE153	2,2´,4,4´,5,5´-hexabromodiphenyl ether
PBDE154	2,2´,4,4´,5,6´-hexabromodiphenyl ether
PBDE183	2,2´,3,4,4´,5´,6-heptabromodiphenyl ether
PBDE209	decabromodiphenyl ether
PBB153	2,2´,4,4´,5,5´-hexabromobiphenyl
