# Supplementary material for: Age, Gender, and BMI Modulate the Hepatotoxic Effects of Brominated Flame Retardant Exposure in US Adolescents and Adults: A Comprehensive Analysis of Liver Injury Biomarkers
Source: Toxics. 2024 Jul 15;12(7):509. doi: 10.3390/toxics12070509 (PMC11280492; doi:10.3390/toxics12070509)
Supplement: Supplementary file 1 [file toxics-12-00509-s001.zip › Table S10.pdf]

Table S10 Qgcomp modeling to assess the associations between combined exposure to serum BFRs and indicators of liver function stratified by age.

	Age: 12-19 (years)		Age: 20-59 (years)		Age: ≥60 (years)	
	β (95%CI)	*P*	β (95%CI)	*P*	β (95%CI)	*P*
AST	−0006 (−0.024, 0.013)	0.551	0.021 (0.007, 0.035)	0.004	0.025 (0.009, 0.041)	0.002
ALT	−0.005 (−0.031, 0.021)	0.724	0.026 (0.008, 0.044)	0.005	0.020 (−0.002, 0.041)	0.071
GGT	−0.011 (−0.038, 0.015)	0.406	0.092 (0.064, 0.119)	< 0.001	0.023 (−0.015, 0.060)	0.236
ALP	0.090 (0.055, 0.124)	< 0.001	0.025 (0.012, 0.037)	< 0.001	0.001 (−0.018, 0020)	0.915
ALB	−0.011 (−0.015, −0.006)	< 0.001	−0.010 (−0.013, −0.007)	< 0.001	0.001 (−0.004, 0006)	0.739
TP	−0.005 (−0.009, −0.001)	0.017	−0.003 (−0.006, 0.000)	0.018	0.003 (−0.001, 0.007)	0.165
TBIL	0.058 (0.028, 0.088)	< 0.001	0.027 (0.009, 0.045)	0.003	0.023 (0.000, 0.047)	0.048

The model was adjusted gender (male, female), race (Mexican American, Other Hispanic, Non-Hispanic White, Non-Hispanic Black, Other Race - including multi-racial), BMI (< 25 kg/m^2 and ≥ 25 kg/m^2), PIR (<1 and ≥ 1), creatinine (continuous), cotinine (continuous), time of blood draw (morning, afternoon, evening), and six-month time period when surveyed (November 1 through April 30, May 1 through October 31).
