# Supplementary material for: Age, Gender, and BMI Modulate the Hepatotoxic Effects of Brominated Flame Retardant Exposure in US Adolescents and Adults: A Comprehensive Analysis of Liver Injury Biomarkers
Source: Toxics. 2024 Jul 15;12(7):509. doi: 10.3390/toxics12070509 (PMC11280492; doi:10.3390/toxics12070509)
Supplement: Supplementary file 1 [file toxics-12-00509-s001.zip › Table S11 .pdf]

Table S11 Qgcomp modeling to assess the associations between combined exposure to serum BFRs and indicators of liver function stratified by gender.

	Male		Female	
	β (95%CI)	*P*	β (95%CI)	*P*
AST	0.021 (0.003, 0.038)	0.021	0.002 (−0.013, 0.019)	0.678
ALT	0.054 (0.030, 0.077)	< 0.001	0.013 (−0.008, 0.033)	0.223
GGT	0.119 (0.085, 0152)	< 0.001	0.025 (−0.009, 0.058)	0.148
ALP	−0.003 (−0.022, 0.017)	0.786	−0.008 (−0.026, 0.010)	0.382
ALB	−0.007 (−0.011, −0.003)	< 0.001	−0.007 (−0.011, −0.003)	0.001
TP	0.000 (−0.003, 0.004)	0.879	0.001 (−0.003, 0.004)	0.713
TBIL	0.059 (0.037, 0.082)	< 0.001	0.024 (0.003, 0.045)	0.024

The model was adjusted age (continuous), race (Mexican American, Other Hispanic, Non-Hispanic White, Non-Hispanic Black, Other Race - including multi-racial), BMI (< 25 kg/m^2 and ≥ 25 kg/m^2), PIR (<1 and ≥ 1), creatinine (continuous), cotinine (continuous), time of blood draw (morning, afternoon, evening), and six-month time period when surveyed (November 1 through April 30, May 1 through October 31).
