# Supplementary material for: Age, Gender, and BMI Modulate the Hepatotoxic Effects of Brominated Flame Retardant Exposure in US Adolescents and Adults: A Comprehensive Analysis of Liver Injury Biomarkers
Source: Toxics. 2024 Jul 15;12(7):509. doi: 10.3390/toxics12070509 (PMC11280492; doi:10.3390/toxics12070509)
Supplement: Supplementary file 1 [file toxics-12-00509-s001.zip › Table S12 .pdf]

Table S12 Qgcomp modeling to assess the associations between combined exposure to serum BFRs and indicators of liver function stratified by BMI.

	BMI < 25 (kg/m^2)		BMI ≥ 25 (kg/m^2)	
	β (95%CI)	*P*	β (95%CI)	*P*
AST	0.007 (−0.011, 0.026)	0.436	0.021 (0.007, 0.035)	0.003
ALT	0.037 (0.014, 0.059)	0.001	0.045 (0.025, 0.065)	< 0.001
GGT	0.055 (0.021, 0.088)	0.001	0.089 (0.057, 0.120)	< 0.001
ALP	−0.041 (−0.068, −0.013)	0.004	0.001 (−0.014, 0.016)	0.916
ALB	−0.008 (−0.012, −0.003)	< 0.001	−0.005 (−0.008, −0.001)	0.008
TP	0.001 (−0.004, 0.005)	0.807	0.001 (−0.002, 0.004)	0.469
TBIL	0.048 (0.023, 0.073)	< 0.001	0.039 (0.019,0.059)	< 0.001

The model was adjusted gender (male, female), age (continuous), race (Mexican American, Other Hispanic, Non-Hispanic White, Non-Hispanic Black, Other Race-including multi-racial), PIR (<1 and ≥ 1), creatinine (continuous), cotinine (continuous), time of blood draw (morning, afternoon, evening), and six-month time period when surveyed (November 1 through April 30, May 1 through October 31).
