# Supplementary material for: Age, Gender, and BMI Modulate the Hepatotoxic Effects of Brominated Flame Retardant Exposure in US Adolescents and Adults: A Comprehensive Analysis of Liver Injury Biomarkers
Source: Toxics. 2024 Jul 15;12(7):509. doi: 10.3390/toxics12070509 (PMC11280492; doi:10.3390/toxics12070509)
Supplement: Supplementary file 1 [file toxics-12-00509-s001.zip › Table S13 .pdf]

Table S13 Associations between single BFR with LFTs based on survey-weighted regression.

	ln_BFRs	β (95% CI)	*P*
AST	ln_PBDE28	0.016 (0.000, 0.031)	0.044
	ln_PBDE47	0.009 (−0.005, 0.022)	0.203
	ln_PBDE99	0.011 (−0.001, 0.023)	0.073
	ln_PBDE100	0.011 (−0.002, 0.023)	0.102
	ln_PBDE153	0.012 (−0.000, 0.024)	0.051
	ln_PBB153	0.008 (−0.001, 0.017)	0.072
ALT	ln_PBDE28	0.021 (0.001, 0.040)	0.042
	ln_PBDE47	0.014 (−0.002, 0.031)	0.091
	ln_PBDE99	0.012 (−0.003, 0.027)	0.105
	ln_PBDE100	0.016 (−0.001, 0.032)	0.057
	ln_PBDE153	0.020 (0.005, 0.036)	0.009
	ln_PBB153	0.022 (0.009, 0.035)	0.001
GGT	ln_PBDE28	0.043 (0.012, 0.075)	0.008
	ln_PBDE47	0.027 (−0.000, 0.055)	0.053
	ln_PBDE99	0.024 (−0.001, 0.049)	0.056
	ln_PBDE100	0.023 (−0.004, 0.050)	0.098
	ln_PBDE153	0.026 (0.003, 0.049)	0.028
	ln_PBB153	0.049 (0.029, 0.028)	< 0.001
ALP	ln_PBDE28	0.012 (−0.002, 0.025)	0.083
	ln_PBDE47	0.010 (−0.002, 0.021)	0.090
	ln_PBDE99	0.007 (−0.004, 0.019)	0.193
	ln_PBDE100	0.007 (−0.004, 0.019)	0.202
	ln_PBDE153	0.003 (−0.008, 0.015)	0.544
	ln_PBB153	0.003 (−0.008, 0.014)	0.559
ALB	ln_PBDE28	−0.010 (−0.013, −0.006)	< 0.001
	ln_PBDE47	−0.009 (−0.012, −0.006)	< 0.001
	ln_PBDE99	−0.008 (−0.010, −0.005)	< 0.001
	ln_PBDE100	−0.007 (−0.010, −0.004)	< 0.001
	ln_PBDE153	0.000 (−0.002, 0.003)	0.927
	ln_PBB153	−0.004 (−0.006, −0.001)	0.009
TP	ln_PBDE28	0.001 (−0.002, 0.005)	0.419
	ln_PBDE47	0.001 (−0.002, 0.004)	0.413
	ln_PBDE99	0.001 (−0.001, 0.004)	0.435
	ln_PBDE100	0.002 (−0.001, 0.005)	0.250
	ln_PBDE153	0.000 (−0.002, 0.002)	0.901
	ln_PBB153	−0.000 (−0.002, 0.002)	0.902
TBIL	ln_PBDE28	0.090 (0.065, 0.114)	< 0.001
	ln_PBDE47	0.058 (0.040, 0.076)	< 0.001
	ln_PBDE99	0.046 (0.032, 0.061)	< 0.001
	ln_PBDE100	0.055 (0.039, 0.071)	< 0.001
	ln_PBDE153	−0.018 (−0.033, −0.002)	0.028

ln_PBB153	0.030 (0.017, 0.043)	< 0.001

The model was adjusted by gender (male, female), age (continuous), race (Mexican American, Other Hispanic, Non-Hispanic White, Non-Hispanic Black, Other Race - including multi-racial), BMI (< 25 kg/m^2 and ≥ 25 kg/m^2), PIR (<1 and ≥ 1), drinking status (never, former, current), creatinine (continuous), cotinine (continuous), time of blood draw (morning, afternoon, evening), and six-month time period when surveyed (November 1 through April 30, May 1 through October 31).
