# Supplementary material for: Age, Gender, and BMI Modulate the Hepatotoxic Effects of Brominated Flame Retardant Exposure in US Adolescents and Adults: A Comprehensive Analysis of Liver Injury Biomarkers
Source: Toxics. 2024 Jul 15;12(7):509. doi: 10.3390/toxics12070509 (PMC11280492; doi:10.3390/toxics12070509)
Supplement: Supplementary file 1 [file toxics-12-00509-s001.zip › Table S14 .pdf]

Table S14 WQS and Qgcomp models to assess the association between combined exposure to serum BFRs and LFTs.

	WQS				Qgcomp	
	Positive		Negative			
	β (95%CI)	*P*	β (95%CI)	*P*	β (95%CI)	*P*
AST	0.014 (0.002, 0.026)	0.018	0.010 (0.001, 0.019)	0.028	0.019 (0.009, 0.029)	< 0.001
ALT	0.037 (0.020, 0.053)	< 0.001	0.019 (0.007, 0.030)	0.001	0.036 (0.023, 0.049)	< 0.001
GGT	0.060 (0.036, 0.085)	< 0.001	--		0.071 (0.052, 0.091)	< 0.001
ALP	0.005 (−0.006, 0.016)	0.333	0.007 (−0.003, 0.017)	0.186	0.005 (-0.005, 0.015)	0.317
ALB	0.001 (−0.001, 0.003)	0.386	−0.010 (−0.012, −0.007)	< 0.001	−0.006 (−0.009, −0.004)	< 0.001
TP	−0.000 (−0.002, 0.002)	0.819	0.000 (−0.002, 0.003)	0.724	0.000 (−0.002, 0.002)	0.703
TBIL	0.053 (0.040, 0.066)	< 0.001	--		0.037 (0.023, 0.050)	< 0.001

The model was adjusted by gender (male, female), age (continuous), race (Mexican American, Other Hispanic, Non-Hispanic White, Non-Hispanic Black, Other Race - including multi-racial), BMI (< 25 kg/m^2 and ≥ 25 kg/m^2), PIR (<1 and ≥ 1), drinking status (never, former, current), creatinine (continuous), cotinine (continuous), time of blood draw (morning, afternoon, evening), and six-month time period when surveyed (November 1 through April 30, May 1 through October 31).
