# Supplementary material for: Age, Gender, and BMI Modulate the Hepatotoxic Effects of Brominated Flame Retardant Exposure in US Adolescents and Adults: A Comprehensive Analysis of Liver Injury Biomarkers
Source: Toxics. 2024 Jul 15;12(7):509. doi: 10.3390/toxics12070509 (PMC11280492; doi:10.3390/toxics12070509)
Supplement: Supplementary file 1 [file toxics-12-00509-s001.zip › Table S2 .pdf]

Table S2 The Spearman rank correlation coefficients for the concentrations of the BFRs.

	ln_PBDE28	ln_PBDE47	ln_PBDE99	ln_PBDE100	ln_PBDE153	ln_PBB153
ln_PBDE28	1.00					
ln_PBDE47	0.87**	1.00				
ln_PBDE99	0.79**	0.93**	1.00			
ln_PBDE100	0.80**	0.90**	0.90**	1.00		
ln_PBDE153	0.40**	0.40**	0.44**	0.59**	1.00	
ln_PBB153	0.38**	0.18**	0.17**	0.20**	0.24**	1.00

** means $P < 0.01$.
