# Supplementary material for: Age, Gender, and BMI Modulate the Hepatotoxic Effects of Brominated Flame Retardant Exposure in US Adolescents and Adults: A Comprehensive Analysis of Liver Injury Biomarkers
Source: Toxics. 2024 Jul 15;12(7):509. doi: 10.3390/toxics12070509 (PMC11280492; doi:10.3390/toxics12070509)
Supplement: Supplementary file 1 [file toxics-12-00509-s001.zip › Table S3 .pdf]

Table S3 Associations between single BFRs and AST levels based on survey-weighted regression.

ln_BFRs		β (95% CI)	*P*
ln_PBDE28	Continuous	0.013 (0.001, 0.026)	0.040
	Categorical		
	≤ 1.504	Reference	
	1.505-1.899	0.015 (−0.007, 0.037)	0.176
	1.900-2.333	0.029 (0.008, 0.051)	0.009
	> 2.333	0.020 (−0.001, 0.042)	0.067
	P trend	0.043	
ln_PBDE47	Continuous	0.008 (−0.003, 0.019)	0.163
	Categorical		
	≤ 4.359	Reference	
	4.360-4.787	0.014 (−0.010, 0.038)	0.249
	4.788-5.287	0.003 (−0.019, 0.025)	0.793
	> 5.287	0.018 (−0.004, 0.039)	0.115
	P trend	0.198	
ln_PBDE99	Continuous	0.009 (−0.001, 0.019)	0.086
	Categorical		
	≤ 2.682	Reference	
	2.683-3.120	0.004 (−0.019, 0.027)	0.742
	3.121-3.666	0.016 (−0.005, 0.038)	0.139
	> 3.666	0.011 (−0.010, 0.032)	0.303
	P trend	0.210	
ln_PBDE100	Continuous	0.008 (−0.003, 0.019)	0.150
	Categorical		
	≤ 2.762	Reference	
	2.763-3.184	0.016 (−0.005, 0.037)	0.135
	3.185-3.682	0.001 (−0.019, 0.022)	0.899
	> 3.682	0.016 (−0.007, 0.038)	0.164
	P trend	0.311	
ln_PBDE153	Continuous	0.011 (0.000, 0.021)	0.046
	Categorical		
	≤ 3.571	Reference	
	3.572-4.014	−0.005 (−0.026, 0.016)	0.639
	4.015-4.494	−0.001 (−0.022, 0.019)	0.887
	> 4.494	0.019 (−0.001,0.041)	0.066
	P trend	0.040	
ln_PBB153	Continuous	0.005 (−0.003,0.012)	0.243
	Categorical		
	≤ 1.661	Reference	
	1.662-2.615	0.026 (0.002, 0.049)	0.032
	2.616-3.319	0.040 (0.013, 0.067)	0.004
	> 3.319	0.037 (0.009, 0.064)	0.009

P trend	0.013

The model was adjusted by gender (male, female), age (continuous), race (Mexican American, Other Hispanic, Non-Hispanic White, Non-Hispanic Black, Other race - including multi-racial), BMI (< 25 kg/m^2 and ≥ 25 kg/m^2), PIR (< 1 and ≥ 1), creatinine (continuous), cotinine (continuous), time of blood draw (morning, afternoon, evening), and six-month time period when surveyed (November 1 through April 30, May 1 through October 31).
