# Supplementary material for: Age, Gender, and BMI Modulate the Hepatotoxic Effects of Brominated Flame Retardant Exposure in US Adolescents and Adults: A Comprehensive Analysis of Liver Injury Biomarkers
Source: Toxics. 2024 Jul 15;12(7):509. doi: 10.3390/toxics12070509 (PMC11280492; doi:10.3390/toxics12070509)
Supplement: Supplementary file 1 [file toxics-12-00509-s001.zip › Table S4 .pdf]

Table S4 Associations between single BFRs and ALT levels based on survey-weighted regression.

ln_BFRs		β (95% CI)	*P*
ln_PBDE28	Continuous	0.014 (−0.002, 0.031)	0.092
	Categorical		
	≤ 1.504	Reference	
	1.505-1.899	0.023 (−0.007, 0.053)	0.124
	1.900-2.333	0.046 (0.016, 0.076)	0.003
	> 2.333	0.025 (−0.003, 0.052)	0.080
	P trend	0.045	
ln_PBDE47	Continuous	0.003 (−0.011, 0.017)	0.654
	Categorical		
	≤ 4.359	Reference	
	4.360-4.787	0.019 (−0.013, 0.051)	0.239
	4.788-5.287	0.013 (−0.018, 0.044)	0.419
	> 5.287	0.010 (−0.017, 0.037)	0.457
	P trend	0.595	
ln_PBDE99	Continuous	0.002 (−0.010, 0.015)	0.710
	Categorical		
	≤ 2.682	Reference	
	2.683-3.120	−0.010 (−0.041, 0.022)	0.546
	3.121-3.666	0.026 (−0.002, 0.055)	0.071
	> 3.666	−0.007 (−0.035, 0.021)	0.633
	P trend	0.948	
ln_PBDE100	Continuous	0.007 (−0.007, 0.021)	0.324
	Categorical		
	≤ 2.762	Reference	
	2.763-3.184	0.006 (−0.022, 0.034)	0.673
	3.185-3.682	−0.003 (−0.031, 0.024)	0.806
	> 3.682	0.014 (−0.014, 0.041)	0.317
	P trend	0.395	
ln_PBDE153	Continuous	0.019 (0.006, 0.033)	0.007
	Categorical		
	≤ 3.571	Reference	
	3.572-4.014	0.014 (−0.017, 0.045)	0.378
	4.015-4.494	0.006 (−0.021, 0.033)	0.680
	> 4.494	0.040 (0.011, 0.069)	0.008
	P trend	0.010	
ln_PBB153	Continuous	0.031 (0.019, 0.043)	< 0.001
	Categorical		
	≤ 1.661	Reference	
	1.662-2.615	0.125 (0.093, 0.158)	< 0.001
	2.616-3.319	0.137 (0.103, 0.171)	< 0.001
	> 3.319	0.130 (0.091, 0.169)	< 0.001

P trend	< 0.001

The model was adjusted by gender (male, female), age (continuous), race (Mexican American, Other Hispanic, Non-Hispanic White, Non-Hispanic Black, Other Race - including multi-racial), BMI (< 25 kg/m^2 and ≥ 25 kg/m^2), PIR (< 1 and ≥ 1), creatinine (continuous), cotinine (continuous), time of blood draw (morning, afternoon, evening), and six-month time period when surveyed (November 1 through April 30, May 1 through October 31).
