# Supplementary material for: Age, Gender, and BMI Modulate the Hepatotoxic Effects of Brominated Flame Retardant Exposure in US Adolescents and Adults: A Comprehensive Analysis of Liver Injury Biomarkers
Source: Toxics. 2024 Jul 15;12(7):509. doi: 10.3390/toxics12070509 (PMC11280492; doi:10.3390/toxics12070509)
Supplement: Supplementary file 1 [file toxics-12-00509-s001.zip › Table S5 .pdf]

Table S5 Associations between single BFRs and GGT levels based on survey-weighted regression.

ln_BFRs		β (95% CI)	*P*
ln_PBDE28	Continuous	0.031 (0.003, 0.059)	0.030
	Categorical		
	≤ 1.504	Reference	
	1.505-1.899	0.022 (−0.017, 0.060)	0.262
	1.900-2.333	0.055 (0.012, 0.098)	0.013
	> 2.333	0.048 (0.004, 0.092)	0.031
	P trend	0.016	
ln_PBDE47	Continuous	0.009 (−0.015, 0.034)	0.453
	Categorical		
	≤ 4.359	Reference	
	4.360-4.787	0.029 (−0.011, 0.068)	0.150
	4.788-5.287	−0.007 (−0.046, 0.032)	0.714
	> 5.287	0.030 (−0.015, 0.075)	0.195
	P trend	0.406	
ln_PBDE99	Continuous	0.009 (−0.013, 0.031)	0.403
	Categorical		
	≤ 2.682	Reference	
	2.683-3.120	−0.008 (−0.048, 0.032)	0.690
	3.121-3.666	0.018 (−0.018, 0.054)	0.327
	> 3.666	0.008 (−0.037, 0.052)	0.733
	P trend	0.552	
ln_PBDE100	Continuous	0.010 (−0.014, 0.035)	0.383
	Categorical		
	≤ 2.762	Reference	
	2.763-3.184	0.016 (−0.020, 0.053)	0.374
	3.185-3.682	−0.007 (−0.044, 0.029)	0.684
	> 3.682	0.023 (−0.019, 0.065)	0.273
	P trend	0.441	
ln_PBDE153	Continuous	0.025 (0.005, 0.046)	0.016
	Categorical		
	≤ 3.571	Reference	
	3.572-4.014	0.005 (−0.038, 0.047)	0.823
	4.015-4.494	−0.006 (−0.049, 0.036)	0.770
	> 4.494	0.045 (0.004, 0.087)	0.034
	P trend	0.041	
ln_PBB153	Continuous	0.059 (0.042, 0.076)	< 0.001
	Categorical		
	≤ 1.661	Reference	
	1.662-2.615	0.167 (0.128, 0.205)	< 0.001
	2.616-3.319	0.223 (0.172, 0.273)	< 0.001
	> 3.319	0.242 (0.185, 0.299)	< 0.001

P trend	< 0.001

The model was adjusted by gender (male, female), age (continuous), race (Mexican American, Other Hispanic, Non-Hispanic White, Non-Hispanic Black, Other Race - including multi-racial), BMI (< 25 kg/m^2 and ≥ 25 kg/m^2), PIR (< 1 and ≥ 1), creatinine (continuous), cotinine (continuous), time of blood draw (morning, afternoon, evening), and six-month time period when surveyed (November 1 through April 30, May 1 through October 31).
