# Supplementary material for: Age, Gender, and BMI Modulate the Hepatotoxic Effects of Brominated Flame Retardant Exposure in US Adolescents and Adults: A Comprehensive Analysis of Liver Injury Biomarkers
Source: Toxics. 2024 Jul 15;12(7):509. doi: 10.3390/toxics12070509 (PMC11280492; doi:10.3390/toxics12070509)
Supplement: Supplementary file 1 [file toxics-12-00509-s001.zip › Table S6 .pdf]

Table S6 Associations between single BFRs and ALP levels based on survey-weighted regression.

ln_BFRs		β (95% CI)	*P*
ln_PBDE28	Continuous	0.038 (0.023, 0.053)	< 0.001
	Categorical		
	≤ 1.504	Reference	
	1.505-1.899	0.017 (−0.014, 0.049)	0.275
	1.900-2.333	0.018 (−0.006, 0.043)	0.143
	> 2.333	0.057 (0.031, 0.084)	< 0.001
	P trend	< 0.001	
ln_PBDE47	Continuous	0.063 (0.051, 0.075)	< 0.001
	Categorical		
	≤ 4.359	Reference	
	4.360-4.787	0.039 (0.011, 0.067)	0.006
	4.788-5.287	0.065 (0.040, 0.091)	< 0.001
	> 5.287	0.122 (0.099, 0.145)	< 0.001
	P trend	< 0.001	
ln_PBDE99	Continuous	0.047 (0.036, 0.058)	< 0.001
	Categorical		
	≤ 2.682	Reference	
	2.683-3.120	0.026 (−0.004, 0.055)	0.084
	3.121-3.666	0.049 (0.021, 0.077)	0.001
	> 3.666	0.095 (0.070, 0.121)	< 0.001
	P trend	< 0.001	
ln_PBDE100	Continuous	0.046 (0.033, 0.058)	< 0.001
	Categorical		
	≤ 2.762	Reference	
	2.763-3.184	0.028 (0.000, 0.056)	0.049
	3.185-3.682	0.053 (0.030, 0.075)	< 0.001
	> 3.682	0.080 (0.056, 0.105)	< 0.001
	P trend	< 0.001	
ln_PBDE153	Continuous	0.021 (0.007, 0.034)	0.003
	Categorical		
	≤ 3.571	Reference	
	3.572-4.014	0.023 (−0.002, 0.048)	0.066
	4.015-4.494	0.033 (0.009, 0.056)	0.007
	> 4.494	0.041 (0.014, 0.068)	0.003
	P trend	0.003	
ln_PBB153	Continuous	−0.041 (−0.054, −0.028)	< 0.001
	Categorical		
	≤ 1.661	Reference	
	1.662-2.615	−0.167 (−0.197, −0.136)	< 0.001
	2.616-3.319	−0.112 (−0.144, −0.081)	< 0.001
	> 3.319	−0.128 (−0.167, −0.089)	< 0.001

P trend	< 0.001

The model was adjusted by gender (male, female), age (continuous), race (Mexican American, Other Hispanic, Non-Hispanic White, Non-Hispanic Black, Other Race - including multi-racial), BMI (< 25 kg/m^2 and ≥ 25 kg/m^2), PIR (< 1 and ≥ 1), creatinine (continuous), cotinine (continuous), time of blood draw (morning, afternoon, evening), and six-month time period when surveyed (November 1 through April 30, May 1 through October 31).
