# Supplementary material for: Age, Gender, and BMI Modulate the Hepatotoxic Effects of Brominated Flame Retardant Exposure in US Adolescents and Adults: A Comprehensive Analysis of Liver Injury Biomarkers
Source: Toxics. 2024 Jul 15;12(7):509. doi: 10.3390/toxics12070509 (PMC11280492; doi:10.3390/toxics12070509)
Supplement: Supplementary file 1 [file toxics-12-00509-s001.zip › Table S7 .pdf]

Table S7 Associations between single BFRs and ALB levels based on survey-weighted regression.

ln_BFRs		β (95% CI)	*P*
ln_PBDE28	Continuous	−0.010 (−0.013, −0.007)	< 0.001
	Categorical		
	≤ 1.504	Reference	
	1.505-1.899	−0.006 (−0.012,0.000)	0.059
	1.900-2.333	−0.010 (−0.016, −0.004)	0.001
	> 2.333	−0.018 (−0.025, −0.012)	< 0.001
	P trend	< 0.001	
ln_PBDE47	Continuous	−0.009 (−0.012, −0.006)	< 0.001
	Categorical		
	≤ 4.359	Reference	
	4.360-4.787	−0.007 (−0.012, −0.001)	0.015
	4.788-5.287	−0.015 (−0.021, −0.008)	< 0.001
	> 5.287	−0.018 (−0.023, −0.012)	< 0.001
	P trend	< 0.001	
ln_PBDE99	Continuous	−0.007 (−0.010, −0.005)	< 0.001
	Categorical		
	≤ 2.682	Reference	
	2.683-3.120	−0.004 (−0.010, 0.002)	0.147
	3.121-3.666	−0.012 (−0.019, −0.005)	0.001
	> 3.666	−0.017 (−0.023, −0.012)	< 0.001
	P trend	< 0.001	
ln_PBDE100	Continuous	−0.008 (−0.011, −0.005)	< 0.001
	Categorical		
	≤ 2.762	Reference	
	2.763-3.184	−0.007 (−0.013, −0.001)	0.019
	3.185-3.682	−0.011 (−0.017, −0.005)	< 0.001
	> 3.682	−0.016 (−0.021, −0.011)	< 0.001
	P trend	< 0.001	
ln_PBDE153	Continuous	0.000 (−0.003, 0.002)	0.680
	Categorical		
	≤ 3.571	Reference	
	3.572-4.014	0.002 (−0.003, 0.007)	0.453
	4.015-4.494	0.003 (−0.002, 0.008)	0.179
	> 4.494	0.000 (−0.004, 0.005)	0.910
	P trend	0.914	
ln_PBB153	Continuous	−0.004 (−0.006, −0.001)	0.005
	Categorical		
	≤ 1.661	Reference	
	1.662-2.615	0.001 (−0.004, 0.006)	0.842
	2.616-3.319	−0.001 (−0.008, 0.006)	0.777
	> 3.319	−0.006 (−0.014, 0.002)	0.161

P trend	0.140

The model was adjusted by gender (male, female), age (continuous), race (Mexican American, Other Hispanic, Non-Hispanic White, Non-Hispanic Black, Other Race - including multi-racial), BMI (< 25 kg/m^2 and ≥ 25 kg/m^2), PIR (< 1 and ≥ 1), creatinine (continuous), cotinine (continuous), time of blood draw (morning, afternoon, evening), and six-month time period when surveyed (November 1 through April 30, May 1 through October 31).
