# Supplementary material for: Age, Gender, and BMI Modulate the Hepatotoxic Effects of Brominated Flame Retardant Exposure in US Adolescents and Adults: A Comprehensive Analysis of Liver Injury Biomarkers
Source: Toxics. 2024 Jul 15;12(7):509. doi: 10.3390/toxics12070509 (PMC11280492; doi:10.3390/toxics12070509)
Supplement: Supplementary file 1 [file toxics-12-00509-s001.zip › Table S8 .pdf]

Table S8 Associations between single BFRs and TP levels based on survey-weighted regression.

ln_BFRs		β (95% CI)	*P*
ln_PBDE28	Continuous	0.000 (−0.003, 0.004)	0.807
	Categorical		
	≤ 1.504	Reference	
	1.505-1.899	−0.002 (−0.008, 0.003)	0.415
	1.900-2.333	0.000 (−0.005, 0.005)	0.987
	> 2.333	0.001 (−0.005, 0.007)	0.821
	P trend	0.619	
ln_PBDE47	Continuous	0.000 (−0.003, 0.002)	0.879
	Categorical		
	≤ 4.359	Reference	
	4.360-4.787	0.001 (−0.003, 0.006)	0.509
	4.788-5.287	−0.003 (−0.007, 0.002)	0.215
	> 5.287	0.000 (−0.005, 0.005)	0.871
	P trend	0.802	
ln_PBDE99	Continuous	0.000 (−0.002, 0.002)	0.887
	Categorical		
	≤ 2.682	Reference	
	2.683-3.120	0.003 (−0.002, 0.007)	0234
	3.121-3.666	−0.001 (−0.005, 0.004)	0.791
	> 3.666	0.000 (−0.005, 0.005)	0.916
	P trend	0.790	
ln_PBDE100	Continuous	0.000 (−0.002, 0.003)	0.759
	Categorical		
	≤ 2.762	Reference	
	2.763-3.184	0.002 (−0.003, 0.007)	0.382
	3.185-3.682	0.001 (−0.004, 0.006)	0.667
	> 3.682	0.001 (−0.003, 0.006)	0.539
	P trend	0.679	
ln_PBDE153	Continuous	0.000 (−0.002, 0.001)	0.593
	Categorical		
	≤ 3.571	Reference	
	3.572-4.014	0.002 (−0.002, 0.006)	0.322
	4.015-4.494	0.002 (−0.003, 0.006)	0.485
	> 4.494	0.001 (−0.003, 0.005)	0.718
	P trend	0.826	
ln_PBB153	Continuous	0.001 (−0.001, 0.002)	0.530
	Categorical		
	≤ 1.661	Reference	
	1.662-2.615	0.006 (0.001, 0.011)	0.013
	2.616-3.319	0.005 (−0.002, 0.011)	0.137
	> 3.319	0.003 (−0.004, 0.010)	0.368

P trend	0.488

The model was adjusted by gender (male, female), age (continuous), race (Mexican American, Other Hispanic, Non-Hispanic White, Non-Hispanic Black, Other Race - including multi-racial), BMI (< 25 kg/m^2 and ≥ 25 kg/m^2), PIR (< 1 and ≥ 1), creatinine (continuous), cotinine (continuous), time of blood draw (morning, afternoon, evening), and six-month time period when surveyed (November 1 through April 30, May 1 through October 31).
