# Supplementary material for: Age, Gender, and BMI Modulate the Hepatotoxic Effects of Brominated Flame Retardant Exposure in US Adolescents and Adults: A Comprehensive Analysis of Liver Injury Biomarkers
Source: Toxics. 2024 Jul 15;12(7):509. doi: 10.3390/toxics12070509 (PMC11280492; doi:10.3390/toxics12070509)
Supplement: Supplementary file 1 [file toxics-12-00509-s001.zip › Table S9 .pdf]

Table S9 Associations between single BFRs and TBIL levels based on survey-weighted regression.

ln_BFRs		β (95% CI)	*P*
ln_PBDE28	Continuous	0.084 (0.063, 0.105)	< 0.001
	Categorical		
	≤ 1.504	Reference	
	1.505-1.899	0.066 (0.031, 0.101)	< 0.001
	1.900-2.333	0.116 (0.085, 0.148)	< 0.001
	> 2.333	0.121 (0.083, 0.158)	< 0.001
	P trend	< 0.001	
ln_PBDE47	Continuous	0.051 (0.036, 0.067)	< 0.001
	Categorical		
	≤ 4.359	Reference	
	4.360-4.787	0.063 (0.034, 0.091)	< 0.001
	4.788-5.287	0.081 (0.057, 0.106)	< 0.001
	> 5.287	0.090 (0.058, 0.122)	< 0.001
	P trend	< 0.001	
ln_PBDE99	Continuous	0.042 (0.029, 0.055)	< 0.001
	Categorical		
	≤ 2.682	Reference	
	2.683-3.120	0.051 (0.020, 0.081)	0.002
	3.121-3.666	0.074 (0.049, 0.098)	< 0.001
	> 3.666	0.084 (0.053, 0.116)	< 0.001
	P trend	< 0.001	
ln_PBDE100	Continuous	0.051 (0.036, 0.065)	< 0.001
	Categorical		
	≤ 2.762	Reference	
	2.763-3.184	0.038 (0.010, 0.067)	0.010
	3.185-3.682	0.069 (0.043, 0.096)	< 0.001
	> 3.682	0.083 (0.054, 0.111)	< 0.001
	P trend	<0.001	
ln_PBDE153	Continuous	−0.019 (−0.034, −0.004)	0.012
	Categorical		
	≤ 3.571	Reference	
	3.572-4.014	−0.017 (−0.044, 0.011)	0.225
	4.015-4.494	−0.038 (−0.065, −0.011)	0.007
	> 4.494	−0.037 (−0.066, −0.008)	0.012
	P trend	0.007	
ln_PBB153	Continuous	0.032 (0.021, 0.043)	< 0.001
	Categorical		
	≤ 1.661	Reference	
	1.662-2.615	0.029 (−0.005, 0.064)	0.095
	2.616-3.319	0.066 (0.030, 0.103)	< 0.001
	> 3.319	0.085 (0.045, 0.126)	< 0.001

P trend	< 0.001

The model was adjusted by gender (male, female), age (continuous), race (Mexican American, Other Hispanic, Non-Hispanic White, Non-Hispanic Black, Other Race - including multi-racial), BMI (< 25 kg/m^2 and ≥ 25 kg/m^2), PIR (< 1 and ≥ 1), creatinine (continuous), cotinine (continuous), time of blood draw (morning, afternoon, evening), and six-month time period when surveyed (November 1 through April 30, May 1 through October 31).
