# Supplementary figures and images for: Age, Gender, and BMI Modulate the Hepatotoxic Effects of Brominated Flame Retardant Exposure in US Adolescents and Adults: A Comprehensive Analysis of Liver Injury Biomarkers
Source: Toxics. 2024 Jul 15;12(7):509. doi: 10.3390/toxics12070509 (PMC11280492; doi:10.3390/toxics12070509)

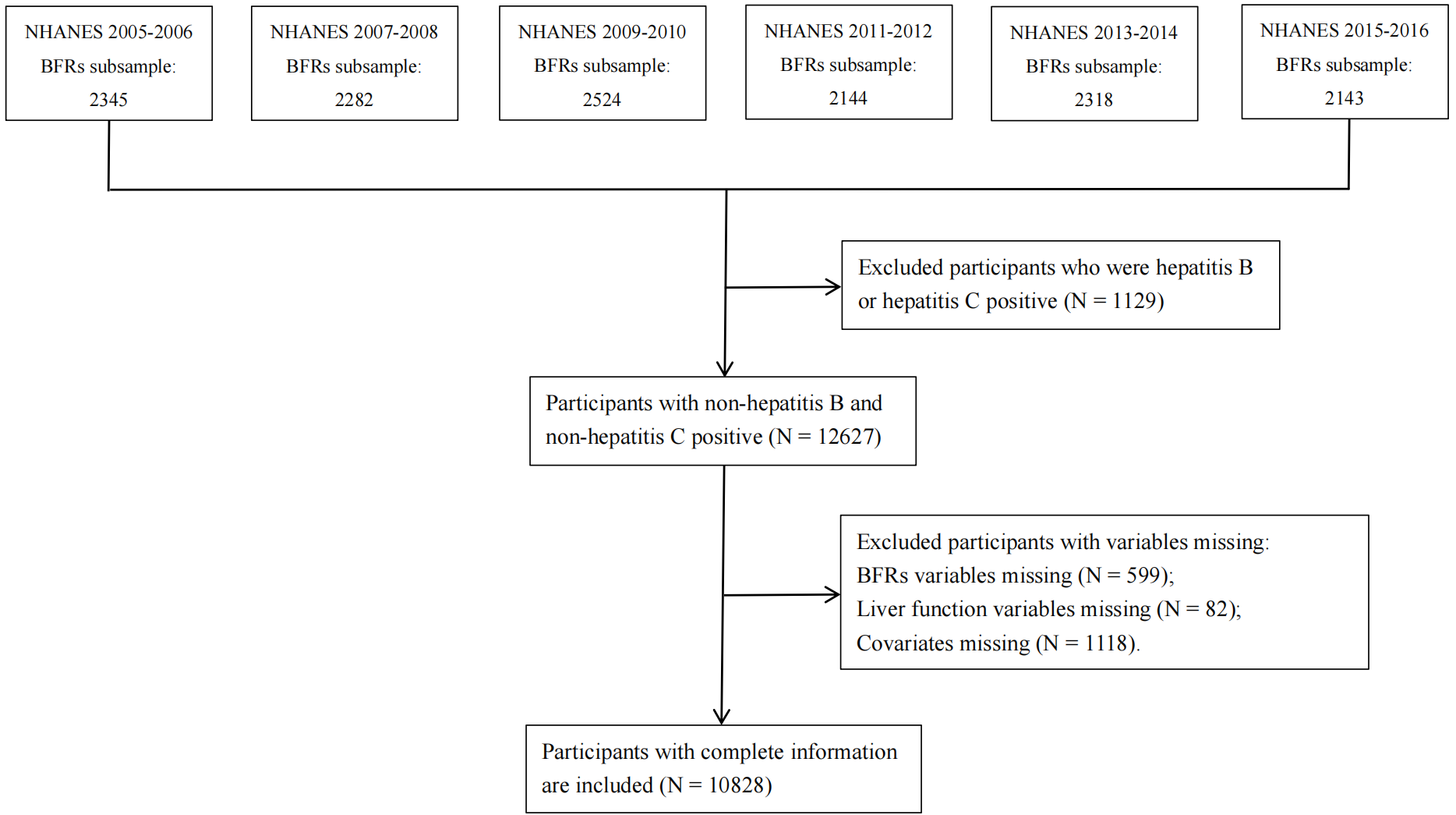

Supplement: Supplementary file 1 [file toxics-12-00509-s001.zip › Figure S1.tif]

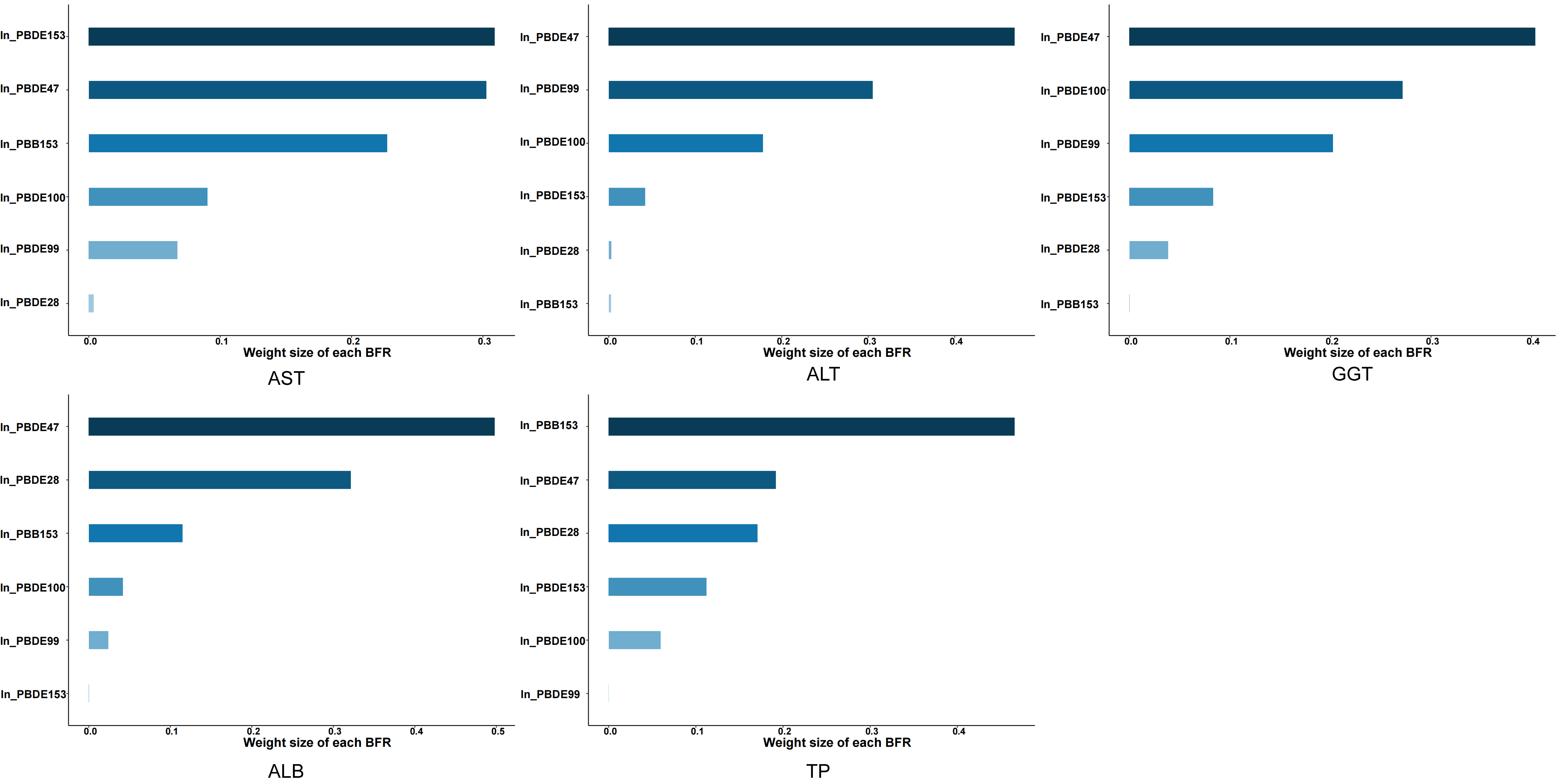

Supplement: Supplementary file 1 [file toxics-12-00509-s001.zip › Figure S10.tif]

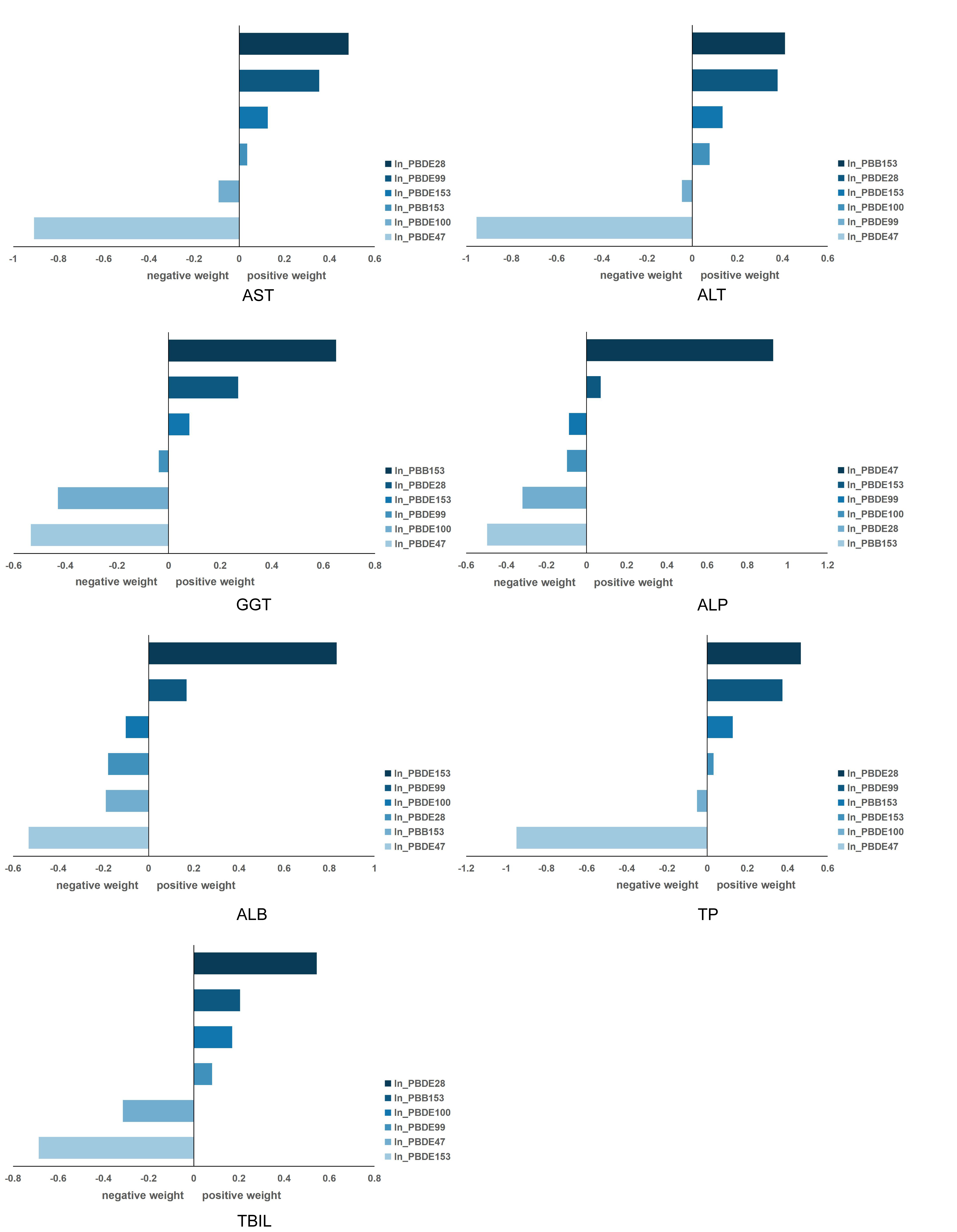

Supplement: Supplementary file 1 [file toxics-12-00509-s001.zip › Figure S11.tif]

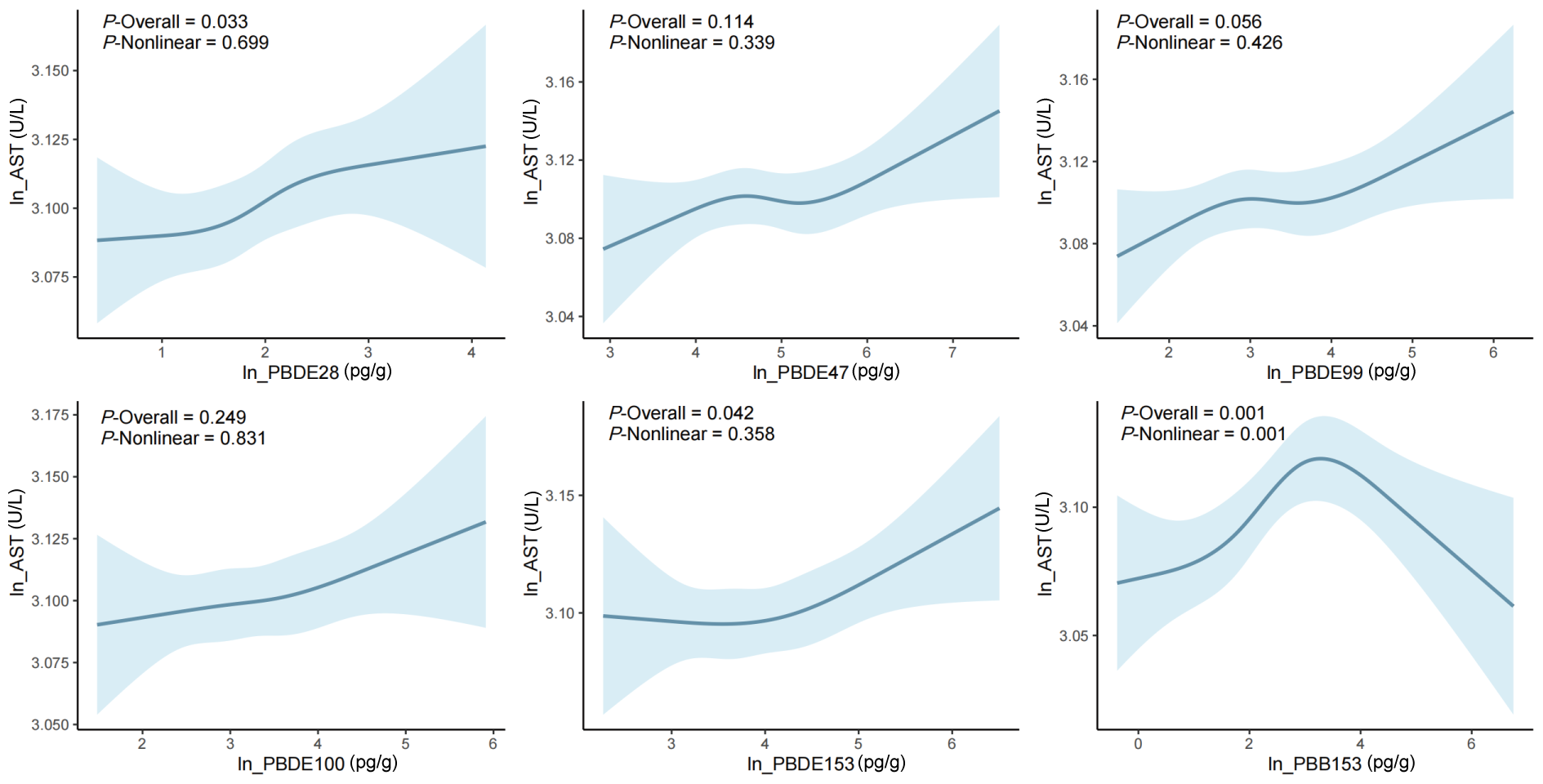

Supplement: Supplementary file 1 [file toxics-12-00509-s001.zip › Figure S2.tif]

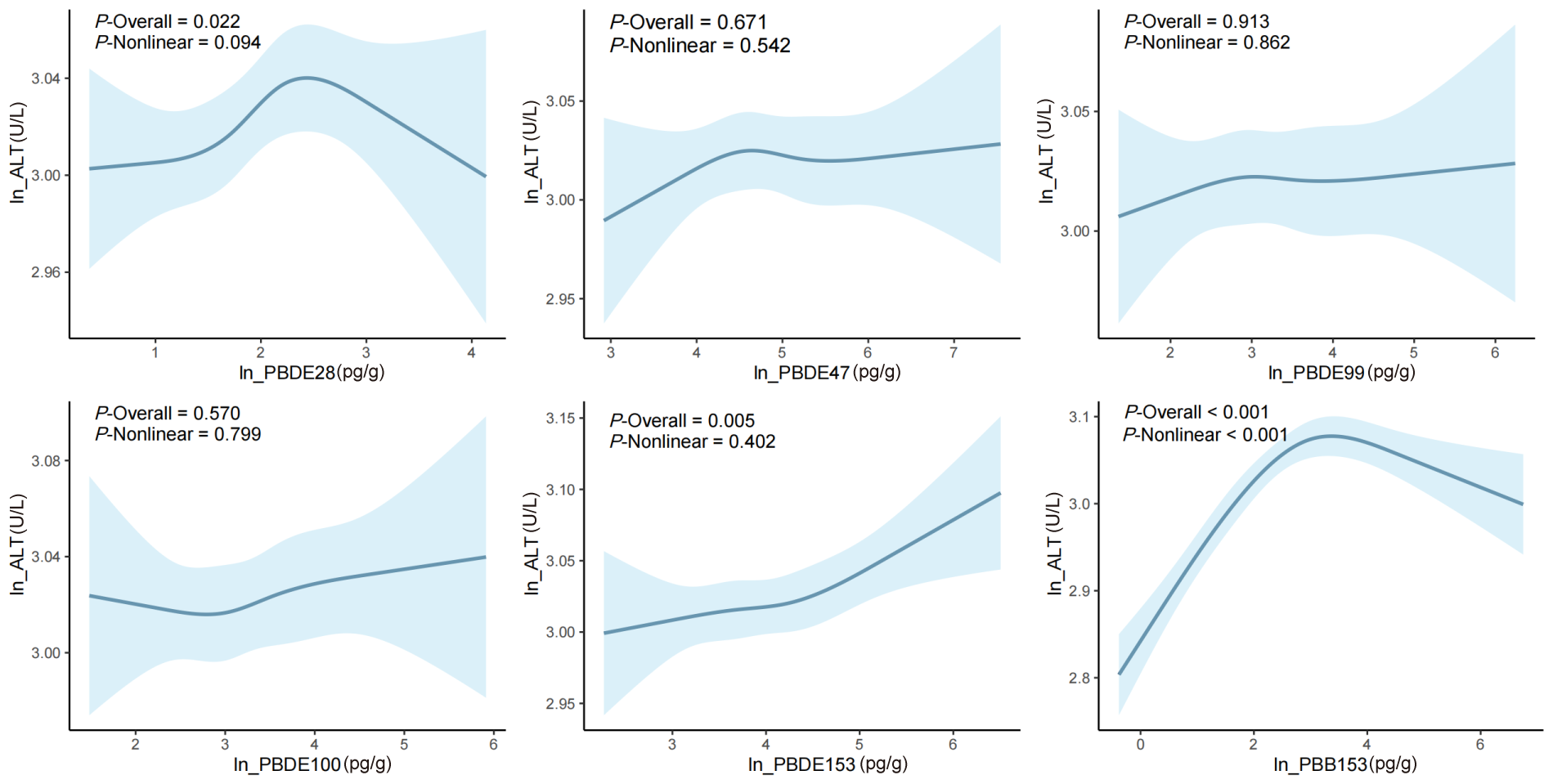

Supplement: Supplementary file 1 [file toxics-12-00509-s001.zip › Figure S3.tif]

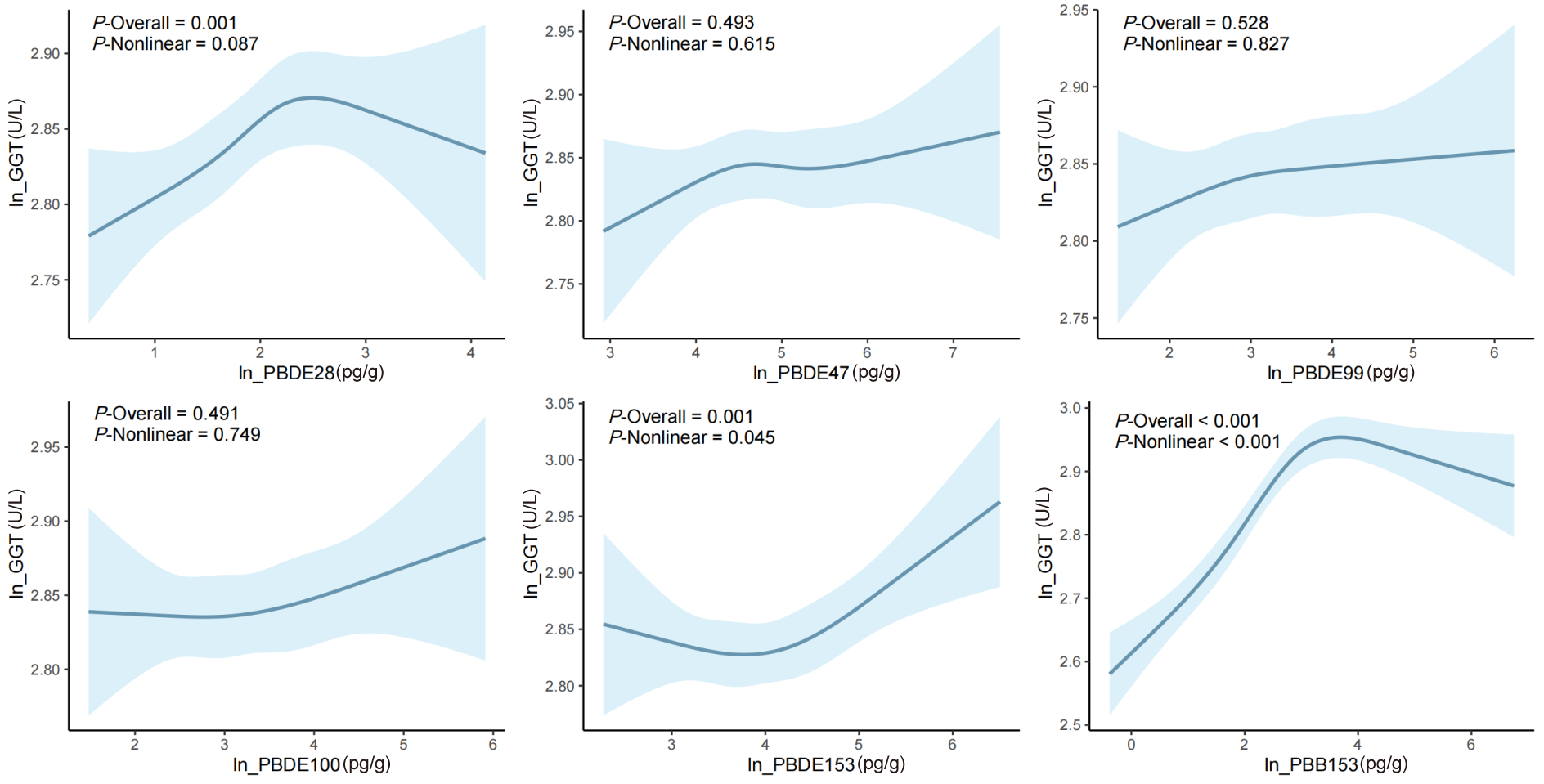

Supplement: Supplementary file 1 [file toxics-12-00509-s001.zip › Figure S4.tif]

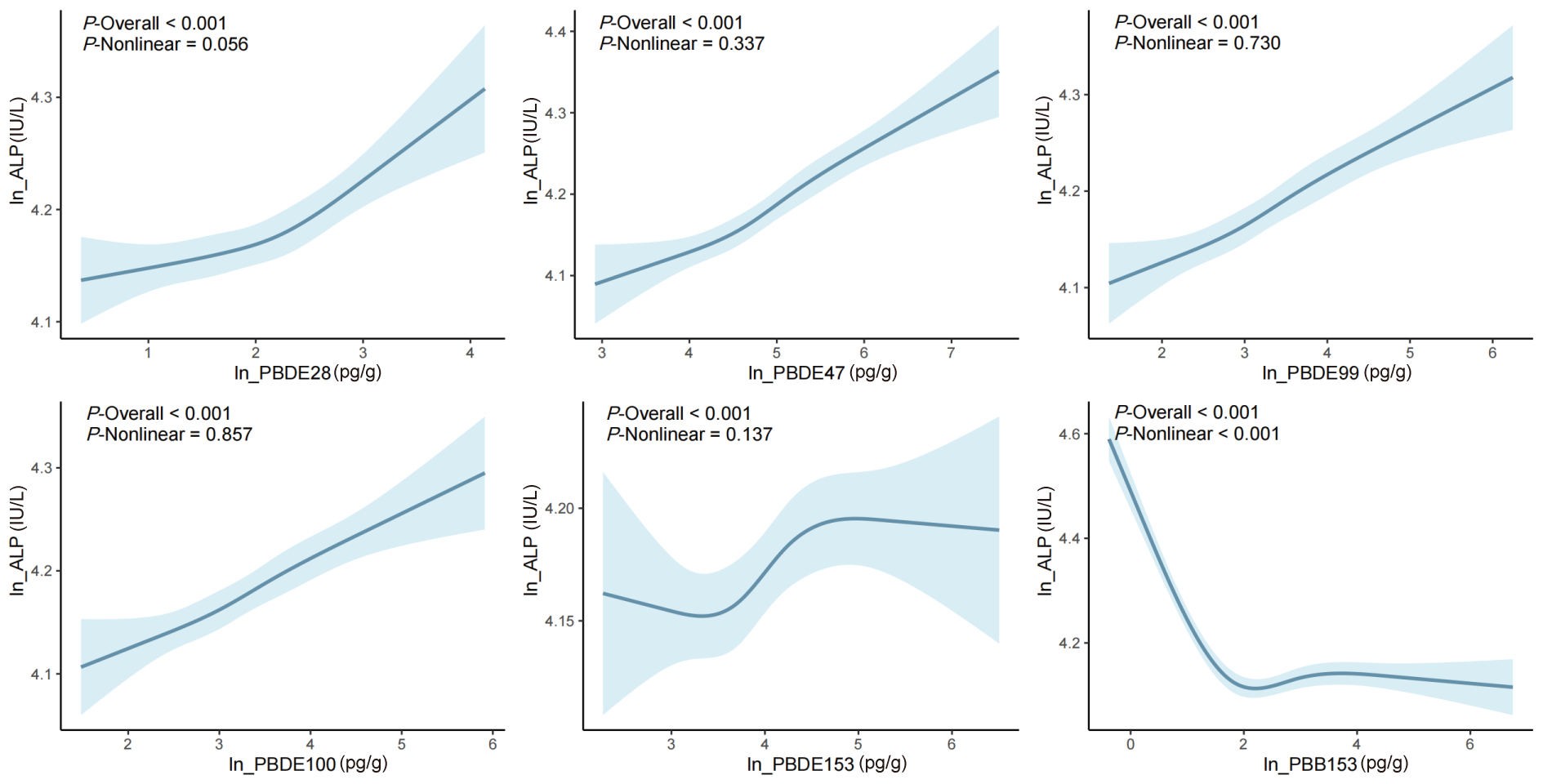

Supplement: Supplementary file 1 [file toxics-12-00509-s001.zip › Figure S5.tif]

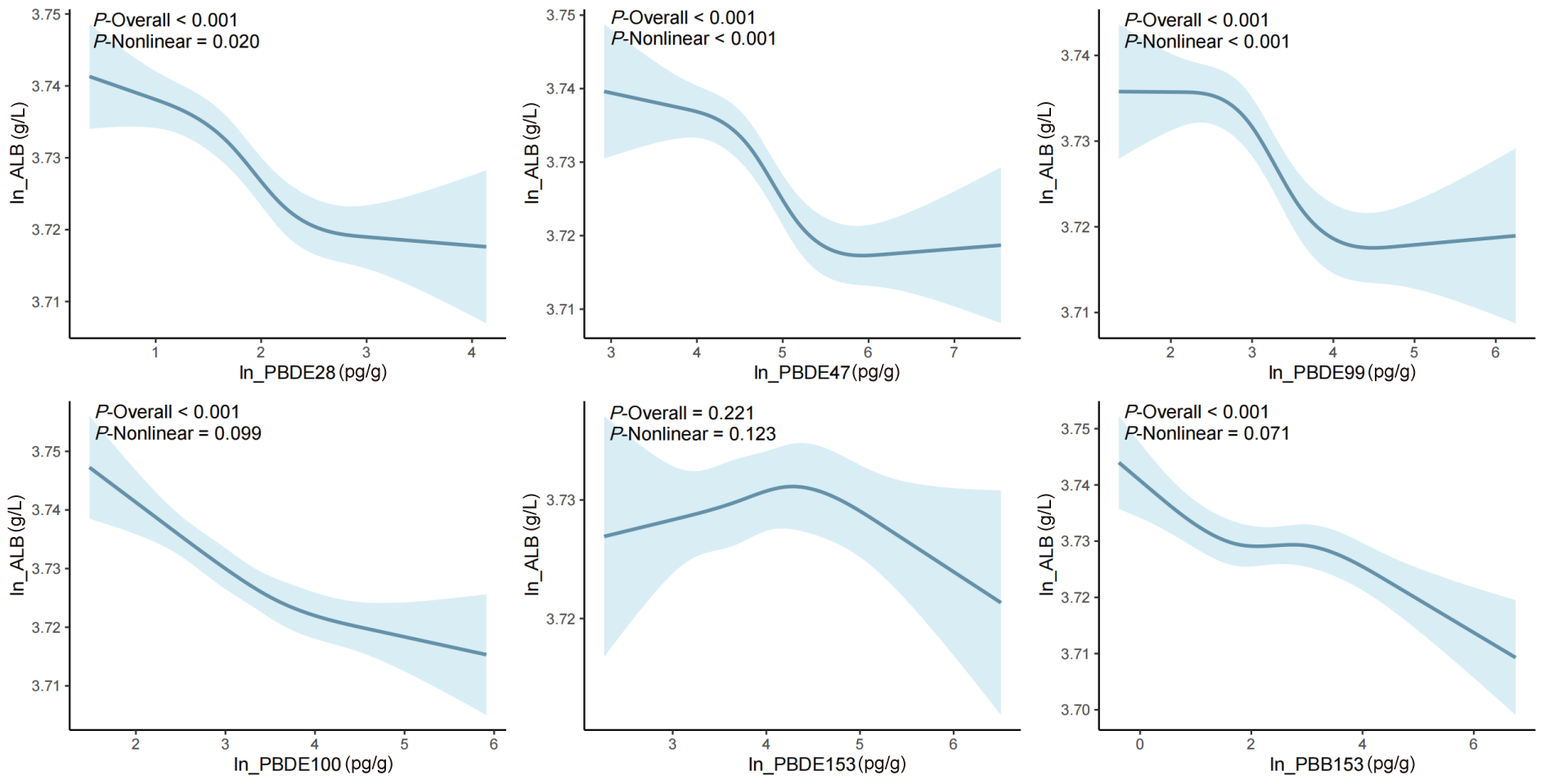

Supplement: Supplementary file 1 [file toxics-12-00509-s001.zip › Figure S6.tif]

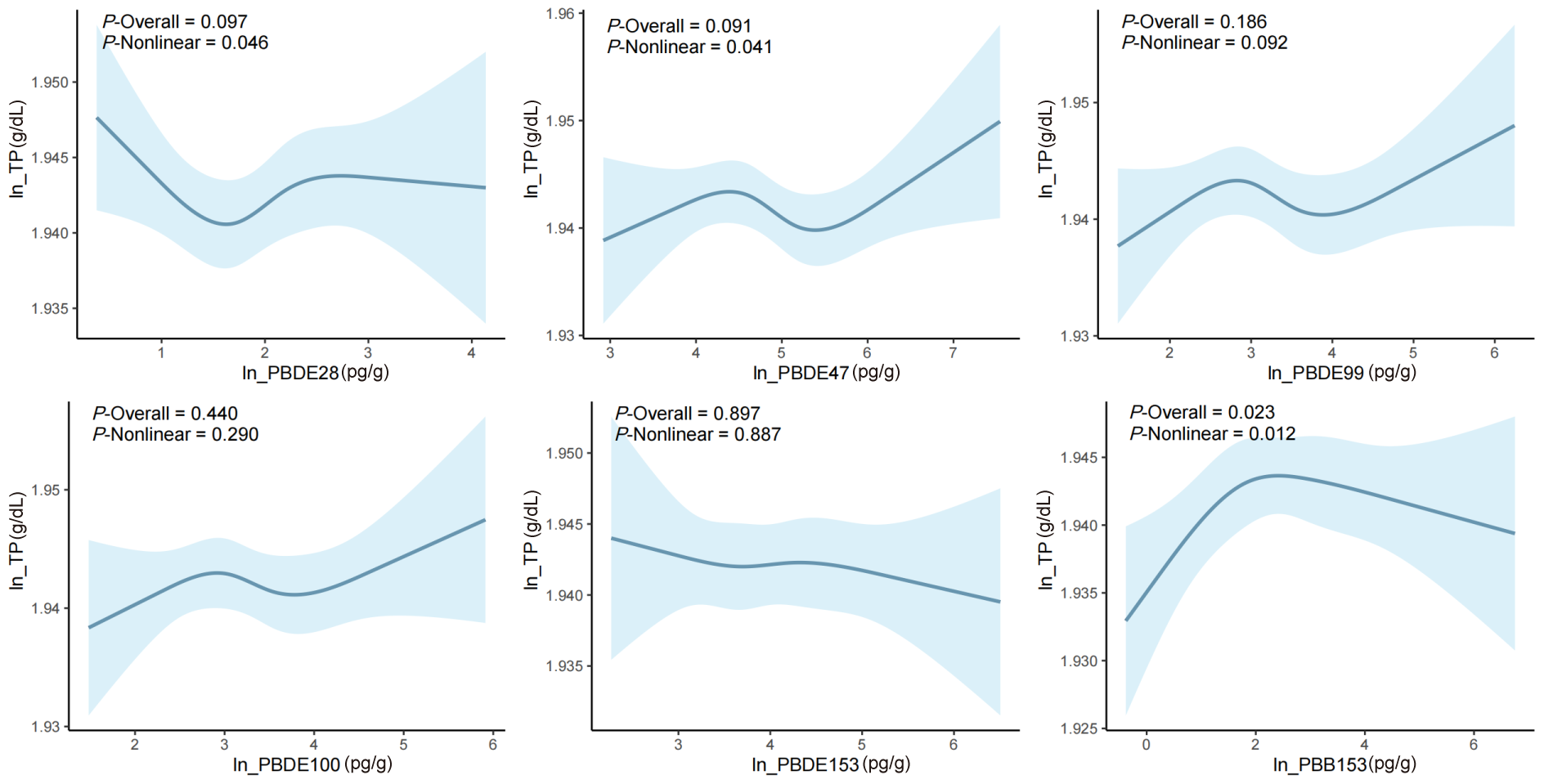

Supplement: Supplementary file 1 [file toxics-12-00509-s001.zip › Figure S7.tif]

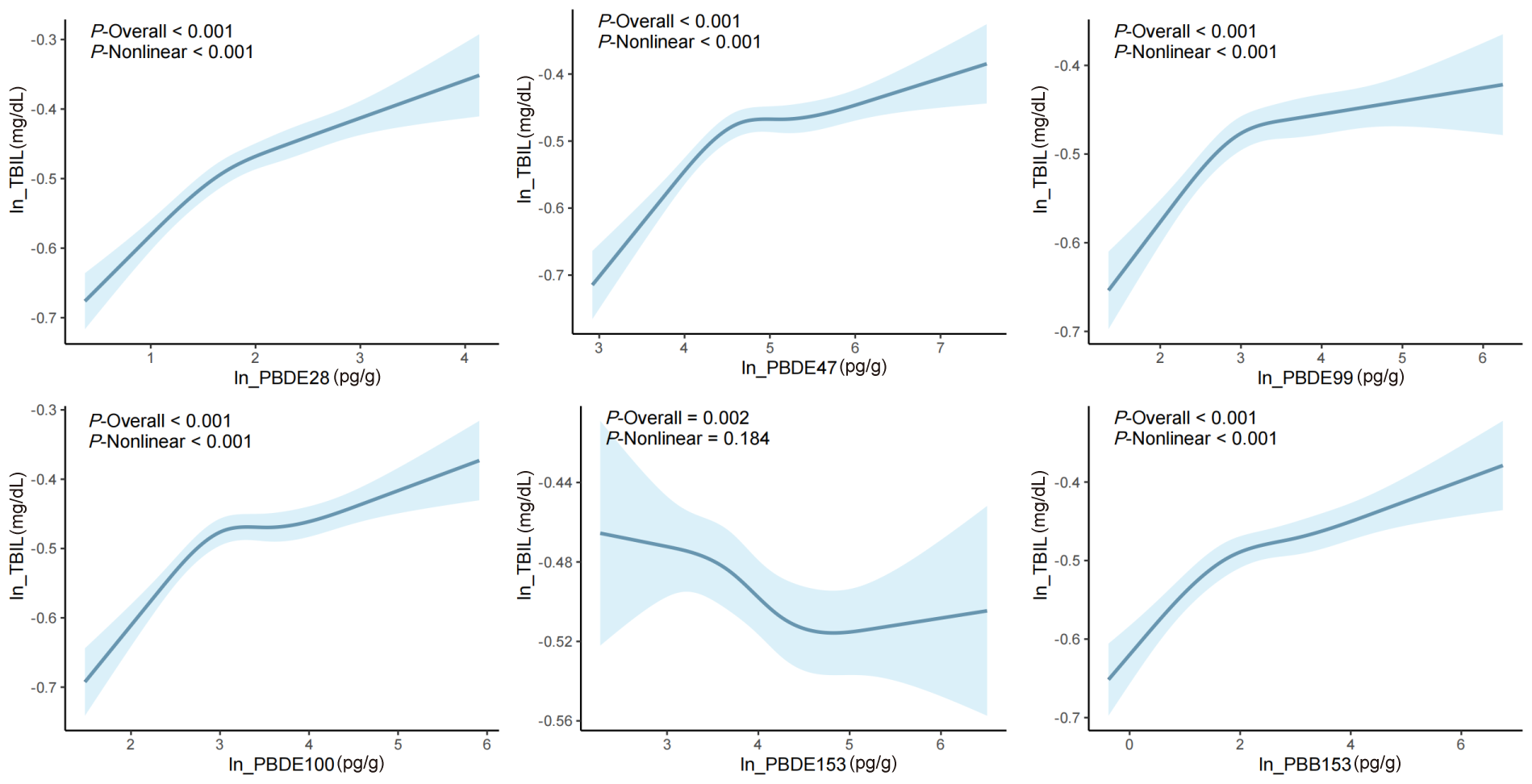

Supplement: Supplementary file 1 [file toxics-12-00509-s001.zip › Figure S8.tif]

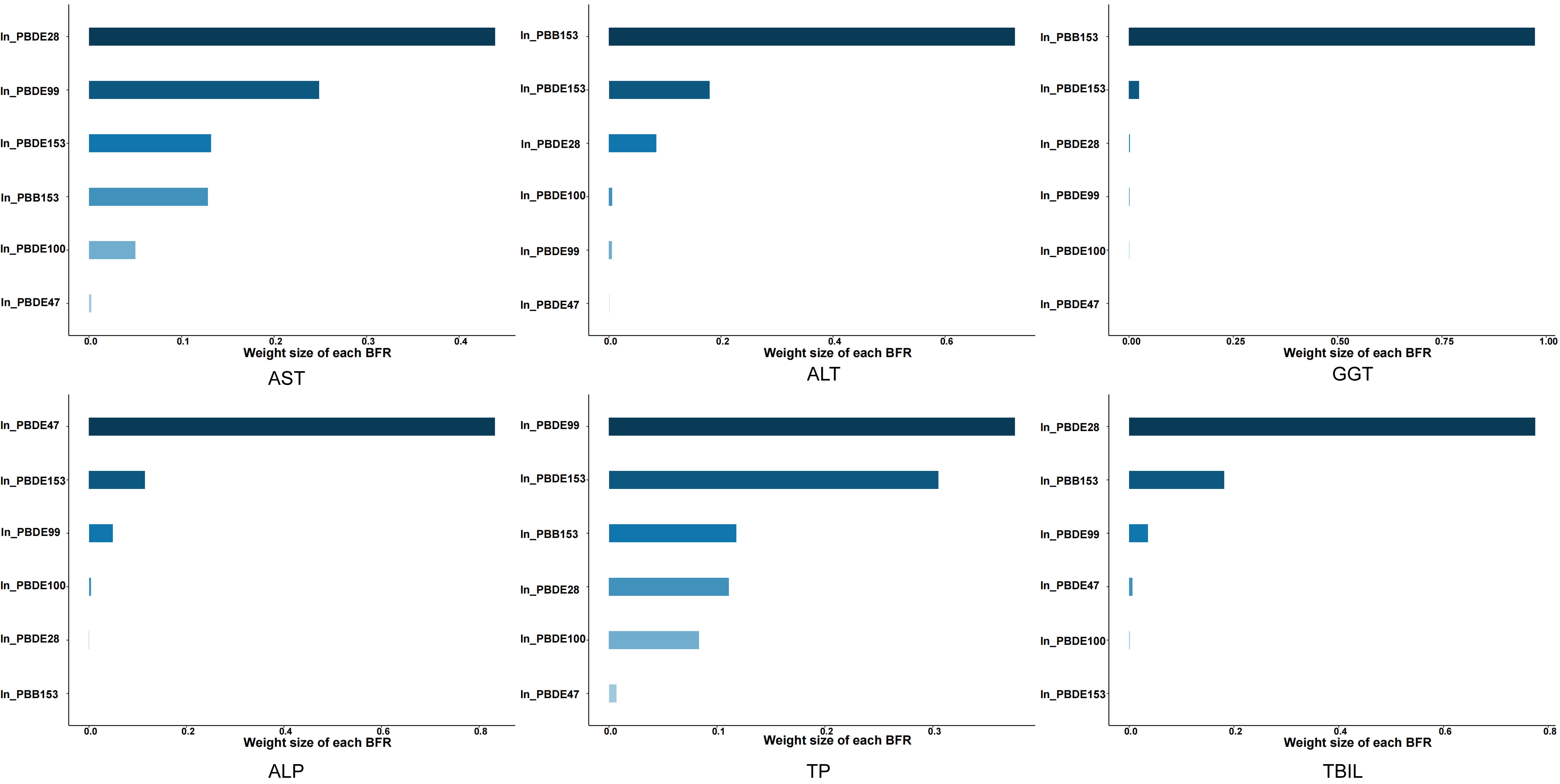

Supplement: Supplementary file 1 [file toxics-12-00509-s001.zip › Figure S9.tif]
